# BK channels of five different subunit combinations underlie the de novo KCNMA1 G375R channelopathy

**DOI:** 10.1085/jgp.202213302

**Published:** 2023-03-30

**Authors:** Yanyan Geng, Ping Li, Alice Butler, Bill Wang, Lawrence Salkoff, Karl L. Magleby

**Affiliations:** 1 Department of Physiology and Biophysics, Miller School of Medicine, University of Miami, Miami, FL, USA; 2 Department of Neuroscience, Washington University St. Louis, St. Louis, MO, USA; 3 Department of Genetics, Washington University St. Louis, St. Louis, MO, USA

## Abstract

The molecular basis of a severe developmental and neurological disorder associated with a de novo G375R variant of the tetrameric BK channel is unknown. Here, we address this question by recording from single BK channels expressed to mimic a G375R mutation heterozygous with a WT allele. Five different types of functional BK channels were expressed: 3% were consistent with WT, 12% with homotetrameric mutant, and 85% with three different types of hybrid (heterotetrameric) channels assembled from both mutant and WT subunits. All channel types except WT showed a marked gain-of-function in voltage activation and a smaller decrease-of-function in single-channel conductance, with both changes in function becoming more pronounced as the number of mutant subunits per tetrameric channel increased. The net cellular response from the five different types of channels comprising the molecular phenotype was a shift of −120 mV in the voltage required to activate half of the maximal current through BK channels, giving a net gain-of-function. The WT and homotetrameric mutant channels in the molecular phenotype were consistent with genetic codominance as each displayed properties of a channel arising from only one of the two alleles. The three types of hybrid channels in the molecular phenotype were consistent with partial dominance as their properties were intermediate between those of mutant and WT channels. A model in which BK channels randomly assemble from mutant and WT subunits, with each subunit contributing increments of activation and conductance, approximated the molecular phenotype of the heterozygous G375R mutation.

## Teaser

A heterozygous BK channelopathy G375R/WT expresses five different types of channels differing in voltage activation and conductance. Four of the five are pathogenic.

## Introduction

**Figure S1.**
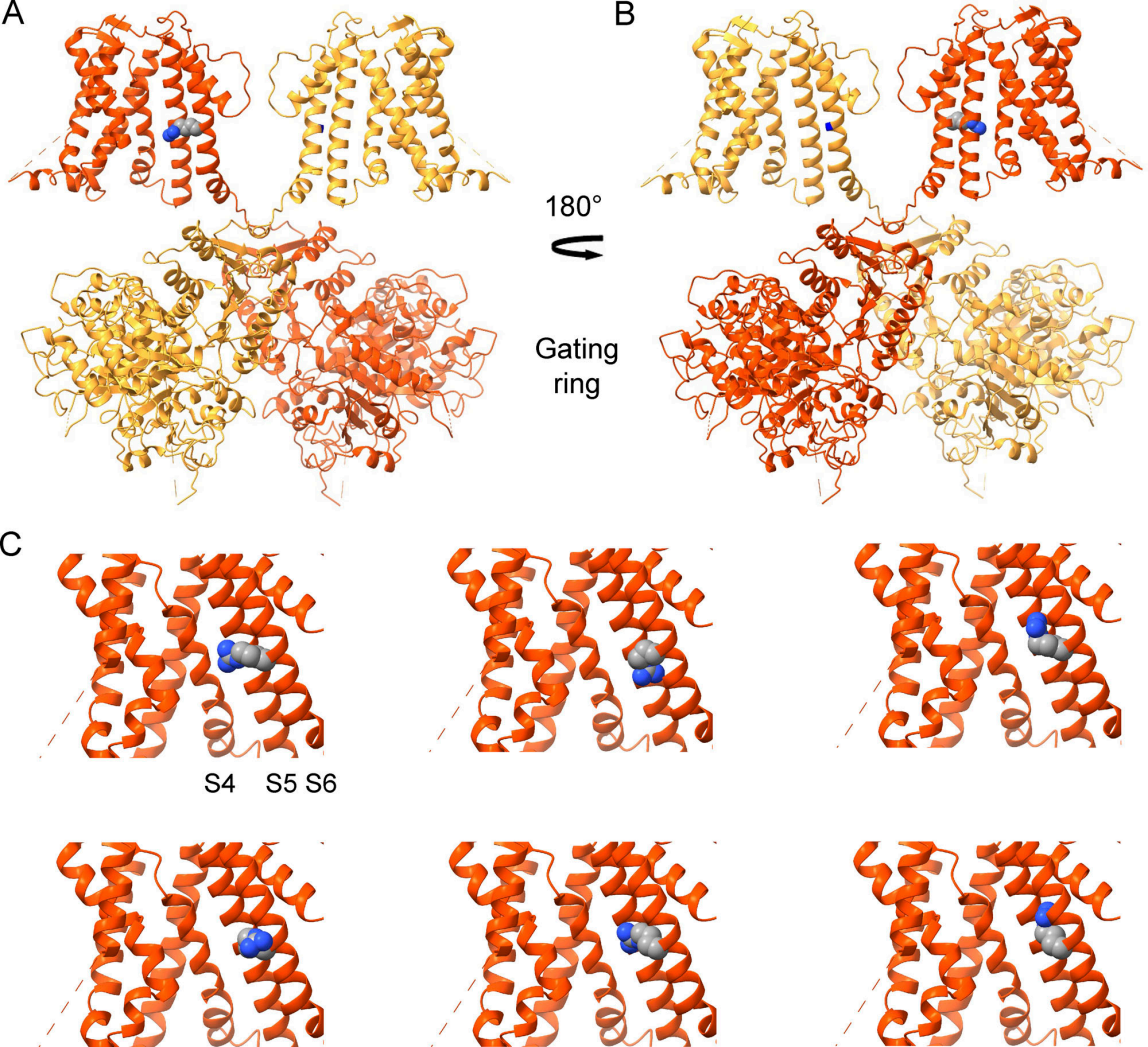
**Cryo-EM ribbon structures of the Ca**^**2+**^**-free human BK channel (PDB ID:** 6V3G; Tao and MacKinnon, 2019**) showing the location of the mutant arginine side chain G375R. (A)** BK channel structure with the front and back pore-forming α subunits removed. The arginine side chain of the G375R mutation is shown in space-filling format on the back side of S6 away from the conduction pathway on the red-colored subunit. Carbon atoms are gray, nitrogen atoms are blue, and hydrogen is not indicated. **(B)** Structure after rotating 180° so that the location (blue mark) of the WT glycine side chain of G375 is visible on the back side of S6 on the yellow subunit. **(C)** Six possible orientations of the mutant arginine side chain are shown, generated by ChimeraX software (www.cgl.ucsf.edu/chimerax). The mutation G375D also produces a left shift in activation (G310D in Chen et al., 2014), but of half the magnitude of G375R (Fig. 3). This raises the possibility that the left shift may be related to the bulk of the substituted side chain rather than the specific charge, which is reversed for these two mutations.

The BK channel (Slo1, KCa1.1) is a large conductance K^+^ selective channel that is synergistically activated by Ca^2+^ and voltage (Barrett et al., 1982; Latorre et al., 1982, 2017; McCobb et al., 1995; Rothberg and Magleby, 2000; Horrigan and Aldrich, 2002; Xia et al., 2002; Salkoff et al., 2006; Horrigan, 2012; Geng and Magleby, 2015; Pantazis and Olcese, 2016; Hite et al., 2017; Tao et al., 2017; Zhou et al., 2017; Tao and MacKinnon, 2019; Geng et al., 2020). BK channels are homotetrameric proteins comprised of four large pore-forming (α) subunits >1,200 amino acids, encoded by the *KCNMA1* gene (Fig. S1). BK channels are widely expressed in many cell types where they modulate smooth muscle contraction (Brenner et al., 2000), transmitter release (Robitaille et al., 1993), circadian rhythms (Harvey et al., 2020), repetitive firing (Gu et al., 2007; Park et al., 2022), and cellular excitability (Montgomery and Meredith, 2012). Mutations in the *KCNMA1* gene that encode the (α) subunit of BK channels are associated with a wide range of diseases, including epilepsy, dyskinesis, autism, multiple congenital abnormalities, developmental delay, intellectual disability, axial hypotonia, ataxia, cerebral and cerebellar atrophy, bone thickening, and tortuosity of arteries (Wang et al., 2009; Yang et al., 2010; Bailey et al., 2019; Liang et al., 2019; Cui, 2021; Miller et al., 2021).

**Figure 1.**
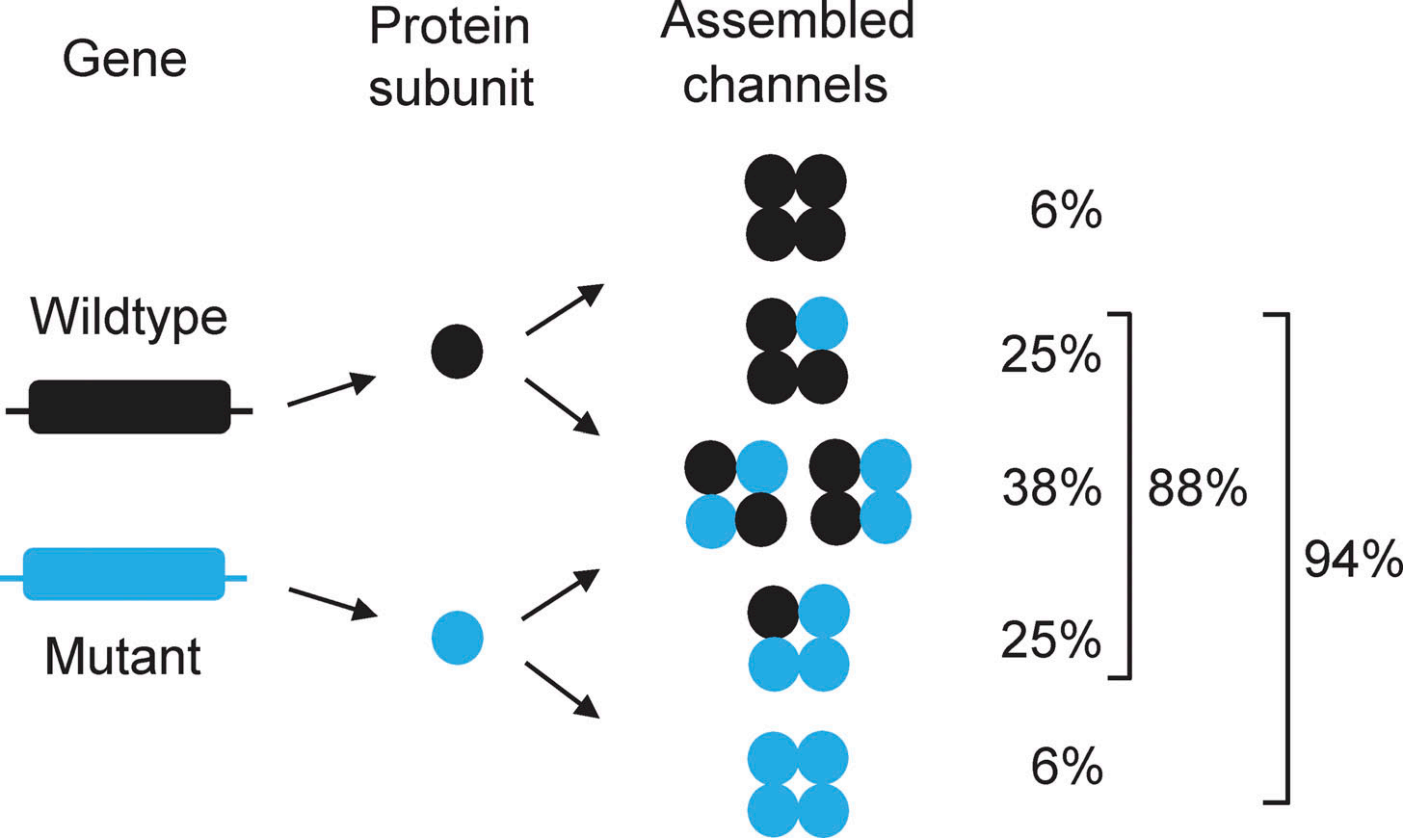
**Protein subunits encoded by WT and mutant alleles for a heterozygous mutation could potentially assemble into five different stoichiometries of tetrameric BK channels and six different subunit arrangements.** See MacKinnon, 1991; Blaine and Ribera, 1998; Ding et al., 1998; Niu and Magleby, 2002; Bergendahl et al., 2019. In this diagram, WT and mutant alleles (gene) encode for WT and mutant protein subunits (protein subunit), which then assemble into tetrameric assembled channels with five different subunit stoichiometries and six different subunit arrangements (assembled channels). The listed theoretical percentages of expression of the five different stoichiometries of assembled channels were calculated with Eq. 2, which assumes that equal numbers of mutant and WT subunits randomly assemble into tetrameric channels, each with equal probability of reaching the surface membrane. With random assembly, ∼6% of the assembled channels are WT comprised of four WT subunits per channel, ∼88% are hybrid (mixed subunit) channels with from one to three mutant subunits per channel, and ∼6% are homotetrameric mutant channels comprised of four mutant subunits per channel. Note that ∼94% of the assembled channels can be pathogenic, as they contain from one to four mutant subunits per channel. The listed percentages of ∼6, ∼38, and ∼94% here and throughout the paper have been rounded from the calculated values of 6.25, 37.5, and 93.75%, respectively. The 37.5% reflects the sum of 25% for hybrid channels with two adjacent mutant subunits and 12.5% for hybrid channels with two diagonal mutant subunits (Bergendahl et al., 2019). For experimentation, assembled channels are those channels expressed following injection of a 1:1 mixture of mutant and WT cRNA into *Xenopus* oocytes to mimic a mutation in which the mutant allele is heterozygous with the WT allele.

Studies of the pathogenic properties of BK channels associated with diseases have often been incomplete, focusing on the homotetrameric mutant channels. Yet, for a mutation heterozygous with the wild-type (WT) allele, mutant and WT subunits have the potential to assemble into five different stoichiometries for tetrameric channels with likely differences in functional properties (Fig. 1; MacKinnon, 1991; Blaine and Ribera, 1998; Niu and Magleby, 2002; Bergendahl et al., 2019; Backwell and Marsh, 2022).

Here, we show that this is the case for a de novo G375R mutation in the (α) subunit of BK channels associated with the Liang-Wang Syndrome (Liang et al., 2019). Three unrelated children who carried this mutation had a syndromic neurodevelopmental disorder associated with severe developmental delay and polymalformation syndrome (Liang et al., 2019). The G375R mutation, located on the back side of the S6 pore-lining helix of the α subunit (Fig. S1), replaces the hydrogen side chain of glycine with a large arginine side chain that might be expected to distort the subunit structure and gating (Chen et al., 2014) as well as add a positive charge that may alter conductance.

To assess the functional effects of a G375R mutation, we recorded currents from whole cells and excised macropatches of the membrane after injecting a 1:1 mixture of G375R mutant and WT cRNA encoding mutant and WT subunits into *Xenopus laevis* oocytes to mimic a de novo mutation heterozygous with a WT allele. When compared with WT, the currents from the 1:1 injection were left-shifted more than −120 mV. This large negative shift in activation indicated a pronounced gain-of-function (GOF) mutation at the cellular level causing the channels to open inappropriately at negative membrane voltages. These observations provide a possible explanation for the severity of the disease associated with the heterozygous G375R mutation.

To investigate the molecular basis underlying the cellular response, detailed single-channel recording (Hamill et al., 1981) following the 1:1 injection suggested that five different types of functional BK channels were expressed: 3% were consistent with WT, 12% with homotetrameric mutant, and 85% with three different types of hybrid (heterotetrameric) channels. All channel types except WT showed a marked GOF in voltage activation and a smaller decrease-of-function (DOF) in single-channel conductance, with both becoming more pronounced as the number of mutant subunits per channel increased. Codominance was observed for the two homotetrameric channels, with homotetrameric WT channels active at the most positive voltage range of channel activity and homotetrameric mutant channels active at the most negative. Partial dominance was observed for the three types of hybrid channels, which were activated at voltages intermediate between those of WT and homotetrameric mutant channels. A model in which BK channels were randomly assembled from mutant and WT subunits, with each subunit contributing increments of activation and conductance, could approximate the molecular phenotype of the heterozygous G375R BK mutation. The possibility that a channelopathy patient with a heterozygous BK channel mutation synthesizes five different types of BK channels in their neurons and other cells, four with aberrant properties, presents a daunting challenge for treatment.

## Materials and methods

### Constructs

Experiments were performed using the human large conductance calcium-activated potassium (BK) channel (hSlo1) KCNMA1 transcript: GenBank accession no. U23767.1 (McCobb et al., 1995) for WT, and a G375R mutation (VFFILGGLAMF to VFFILRGLAMF) was constructed to match the G375R de novo variant described by Liang et al. (2019). G375R is at the same position as G310 in Tao and MacKinnon (2019), which they called the gating hinge residue. The numbering differs because Tao and MacKinnon (2019) used an alternative transcript missing the first 65 amino acids compared to U23767.1. Both our hSlo1 WT cRNA plasmid and WT mammalian transfection plasmid contain the same hSlo1 insert and are described in McCobb et al. (1995). Overlap extension PCR cloning was used to generate the G375R mutation, which was verified by sequencing. The new construct was linearized downstream of the end of coding and transcribed with T3 using Invitrogen’s T3 mMessage mMachine kit to make cRNA for injection into *Xenopus* oocytes. To mimic the effects of a heterozygous mutation, G375R mutant and WT cRNA were mixed in a 1:1 molar ratio before injection into oocytes. For expression in HEK293 cells, two mammalian transfection plasmids were created. DNA fragments containing the complete channel cDNAs of either the WT or G375R mutant and two unique restriction sites flanking it were amplified by PCR and ligated into mammalian transfection plasmids, which were verified by sequencing.

### Expression of BK channels in oocytes for whole-cell recording

Defolliculated *Xenopus* oocytes were injected with 0.5–150 ng of cRNA using a Nanoject II (Drummond Scientific) and incubated at 18°C for 2–5 d before recording. Incubation was in ND96 complete medium consisting of (in mM) 96 NaCl, 2 KCl, 1.8 CaCl_2_, 5 MgCl_2,_ and 5 HEPES, adjusted to pH 7.5, supplemented with 2.5 mM sodium pyruvate and 100 µg/ml each penicillin and streptomycin. The two-microelectrode whole-cell voltage-clamp recordings from oocytes were obtained in ND96 medium with 1 mM added 4,4‘-diisothiocyanatostilbene-2,2’-disulphonic acid disodium salt hydrate (DIDS) to block the endogenous chloride conductance. Currents were obtained with an Oocyte Clamp OC-725C amplifier (Warner Instrument Corp.). Recordings were low-pass filtered at 1 kHz and digitized at 10 kHz. Electrodes were made with borosilicate glass capillaries (World Precision Instruments) pulled with a Sutter Instrument Co. P-87 pipette puller and filled with 3 M KCl.

### Macropatch and single-channel recordings from BK channels expressed in *Xenopus* oocytes

Oocytes were injected with 0.1–18 ng of cRNA and incubated at 18°C for 2–5 d in Barth’s Solution (in mM): 88 NaCl, 1 KCl, 2.4 NaHC0_3_, 0.33 Ca(NO_3_)_2_, 0.41 CaCl_2_, 0.82 MgSO_4_, 15 mM HEPES, pH 7.6, plus 12 µM tetracycline. Macro- and single-channel currents were recorded from inside-out patches of the membrane (Hamill et al., 1981) excised from oocytes at room temperature (21–24°C). pClamp 9.0 software (Molecular Devices) was used to drive an Axopatch 200B amplifier to collect the currents. For macropatch current recordings, borosilicate pipettes with 0.5–2 MΩ resistance were used. The macrocurrents were filtered at 10 kHz and sampled at 100 kHz. A minus P/4 protocol was used to remove capacitive transients and leak currents. For single-channel recordings, borosilicate pipettes with 8–12 MΩ resistance were used. The single-channel currents were filtered at 5 kHz and sampled at 200 KHz. The pipette (external) solution contained (in mM) 160 KCl, 2 MgCl_2_, and 5 TES buffer, pH 7.0. The internal membrane surface of the excised patches was perfused by two different solutions. The designated 0 Ca^2+^ solution had a free Ca^2+^ <0.01 µM and contained (in mM) 160 KCl, 1 EGTA, 1 HEDTA, and 10 HEPES, pH 7.0. The solution with 300 µM internal Ca^2+^ contained (in mM) 160 KCl, 0.3 CaCl_2_, and 10 HEPES, pH 7.0. Procedures to obtain oocytes from *Xenopus* laevis were approved by the University of Miami Animal Care and Use Committee. Macropatch and single-channel recordings were analyzed with Clampfit 10.7 software (Molecular Devices) and SigmaPlot 12.

For injection of only G375R cRNA into *Xenopus* oocytes, we found that it was difficult to get giga-ohm seals of sufficient quality for macropatch recordings using the lower resistance electrodes required for such recordings. Hence, macropatch currents are not presented for injection of only G375R cRNA into oocytes. However, it was still possible to obtain high-quality giga-ohm seals for single-channel recording using the higher resistance pipettes required for such recordings. We also found that the viability of the oocytes was greatly reduced following injection of only G375R cRNA, with the oocytes often starting to die by the second or third day after injection, rather than after a week or more, perhaps because a large fraction of the G375R homotetrameric mutant channels would be expected to be open at resting membrane potentials, as will be shown in later sections. These viability problems were not observed for injection of 1:1 mixtures of G375R mutant and WT cRNA, which gave less pronounced negative shifts in activation, or for injections of only WT cRNA.

The open probability (Po) of each single channel analyzed in detail was determined for a range of voltages with Clampfit 10.7 using 50% threshold analysis to measure open and closed interval durations. Po at each voltage was calculated by dividing the total open time by the sum of the open and closed times. The duration of recordings to estimate Po ranged from ∼5 s to ∼3 min, with the time increasing as the Po decreased. For macropatch current recordings, relative conductance was determined from macroscopic tail current amplitudes using the voltage protocols indicated in the figure legends. G/G_max_ vs. V (G-V) plots for macrocurrent recordings and Po vs. V (Po-V) plots for single channels were fitted with a Boltzmann function to estimate voltage for half activation (*V*_h_), the voltage required for half-maximal activation, and *b*, a measure of voltage sensitivity, using

$$G/G_{\max}=1/\left\{1+\exp\left[\left(V_{h}-V\right)/b\right]\right\}\text{,}$$ (1)

where G/G_max_ is the ratio of conductance to maximum conductance and *b* is the slope factor which gives a measure of voltage sensitivity, where *b* indicates the change in millivolts required to increase G/G_max_ (or Po for single channel recording) e-fold at very low G/G_max_ (or Po). Note that an increase in *b* indicates a decrease in slope and voltage sensitivity.

Single-channel conductance was determined at 100 mV by setting horizontal cursor lines by eye to the open and closed current levels of single-channel recordings.

### HEK293 cell culture

As described previously (Santi et al., 2006), human embryonic kidney HEK293 cells (ATCC CRL-1573; ATCC) were cultured in Gibco Dulbecco’s modified Eagle’s medium (DMEM) with 10% fetal bovine serum, 100 units ml^−1^ penicillin, and 100 μg ml^−1^ streptomycin (Thermo Fisher Scientific) and incubated at 37°C with 5% CO_2_. These cells were grown and passaged twice a week in T25 flasks (MidSci).

### Expression of BK channels in HEK293 cells

HEK293 cells were plated at a density of ∼400,000 cells per 40-mm petri dish (#93040; TPP catalog) a day before transfection. For transfection, plasmid DNA containing cDNA encoding for BK WT and/or BK G375R (mutant) subunits was added to cell layers that were 70–90% confluent using Lipofectamine 2000 transfection reagent (catalog #11668-027; Thermo Fisher Scientific) following manufacturer’s instructions. 2 μg cDNA of WT, mutant, or a mix of WT and mutant (1:1 ratio) was transfected per 40 mm dish. As a marker for transfection, cells were cotransfected with pmaxGFP, a CMV plasmid expressing green fluorescent protein (Amaxa Biosystems) at 0.2 μg per dish. After transfection, the cells were incubated at 37°C with 5% CO_2_ for 2–4 d until recording. The recordings were then performed at room temperature (22°C). Fluorescent cells were used to study the function of the ion channels that the HEK293 cells were transfected with; non-fluorescing cells were used as a non-transfected controls (Han et al., 2004).

### Whole-cell recording from HEK293 cells

Whole-cell recordings were obtained from HEK293 cells using an Axopatch 200B amplifier (Molecular Devices). Recordings were filtered at 5 kHz with the amplifier internal filter and digitized at 50 kHz using a Digidata 1550B digitizer (Molecular Devices). The recording pipette was filled with (in mM) 140 KMES, 5 EGTA, and 10 HEPES, pH 7.4 with KOH. Bath solutions contained (in mM) 135 NaMES, 5 KMES, 1 MgCl_2_, and 10 HEPES, pH 7.4 with NaOH.

### Calculating the percentages of the five different stoichiometries of BK channels that can be expressed when mimicking a heterozygous mutation

As shown in Fig. 1, a mutation heterozygous with WT can potentially produce five different types of channels defined by their stoichiometry. The binomial equation was used to calculate the expected percentages of different combinations of mutant and WT subunits for tetrameric BK channels (Blaine and Ribera, 1998; MacKinnon, 1991). For the calculations in Fig. 1, this approach assumes that WT and mutant alleles produce equal numbers of mutant and WT subunits that assemble randomly into tetrameric channels of different stoichiometries, labeled assembled channels in Fig. 1, and that each assembled channel has the same probability of being expressed, independent of subunit composition, such that

$$F_{i}=\left\{4!/\left[\left(4‐i\right)!i!\right]\right\}F_{\mathrm{WT}}^{\left(4-i\right)}F_{M}^{i}\text{,}$$ (2)

where *F*_*i*_ is the decimal fraction of channels with *i* mutant subunits, *F*_WT_ is the fraction of WT cRNA injected, and *F*_M_ is the fraction of mutant cRNA injected, where *F*_WT_ + *F*_M_ = 1. For BK channels, *i* ranges from 0 to 4, giving five channel types based on subunit composition. Setting both *F*_WT_ and *F*_M_ to 0.5 gives the fractions of the various channel types for a heterozygous mutation. *F*_i_ multiplied by 100% gives the percentage of expressed channels of each type.

### Simulating discrete probability distributions of the number of BK channels of each stoichiometry expected for equal production and random assembly of mutant and WT subunits

Simulated discrete probability distributions were used to determine if the experimentally observed number of channels of each stoichiometry in a group of 33 assembled channels expressed following 1:1 injection of mutant and WT cRNA differed significantly from the numbers expected assuming equal production and a random assembly of mutant and WT subunits. The discrete probability distributions for each of the five types of channels based on subunit stoichiometry were simulated as follows. The first step was to generate a group of 33 channels by assuming a random assembly of mutant and WT subunits drawn from equal numbers of mutant and WT subunits (with replacement) for each of the 33 channels in the group. To assemble each of the 33 channels, four random numbers between 0 and 1 were drawn. Each random number <0.5 indicated a WT subunit and each random number ≥0.5 indicated a mutant subunit. The subunit composition of each simulated channel then indicated its channel type from the stoichiometry. The number of channels of each type in each group of 33 channels was tabulated and binned into five separate frequency histograms, one for each channel type. To give an example for one group of 33 channels, the simulation may have assembled 1 channel with four WT subunits, 10 channels with one mutant and three WT subunits, 11 channels with two mutant and two WT subunits, 7 channels with three mutant and one WT subunits, and 4 channels with four mutant subunits. The frequency histogram indicating the number of WT channels in the groups of 33 channels would then have one count added to the specific bin indicating 1 WT channel; the frequency histogram indicating the number of channels with one mutant and three WT subunits found in groups of 33 channels would then have one count added to the bin indicating 10 channels of that type; the frequency histogram indicating the number of channels with two mutant and two WT subunits found in the group of 33 channels would then have one count added to the bin indicating 11 channels of that type, with similar binning for the 2 remaining channel types. This process was repeated for 10^6^ groups of 33 channels, where one count was added to each of the five frequency histograms for each group of 33 channels. If no channels of a given type were observed in a group of 33 channels, then the count was added to bin 0 to indicate that 0 channels of that type were observed in the group of 33 channels. Each frequency histogram for each channel type then contained 10^6^ total counts. Dividing the number of counts in each bin in the frequency histograms by 10^6^ counts in that frequency histogram gave the discrete probabilities of observing 0, 1, 2, 3…33 channels of the indicated stoichiometry in a group of 33 channels. The discrete probabilities summed to 1.0 for each distribution. The five discrete probability distributions, one for each of the five types of assembled channels, are presented in Fig. S4.

### Software

The increments of single-channel conductance and *V*_h_ added by each G375R mutant and WT subunit for Fig. 5 C and Fig. 6 D were optimized by minimizing the sum of the squared differences between the experimental values and those calculated with the equations in the legends of Fig. 5 C and Fig. 6 D by running Solver in Excel with solving method GRG nonlinear. The computer program for simulating the discrete probability distributions for observing a given number of channels of a given type in a group of 33 channels assuming random assembly of mutant and WT subunits, Binomdouble5-33.bas, was written in QB64 and is available from K.L. Magleby upon request.

### Statistics

Error bars in the figures and error estimates in the text are SEM. Significance was determined with the two-tailed *t* test unless otherwise indicated.

### Online supplemental material

**Figure 2.**
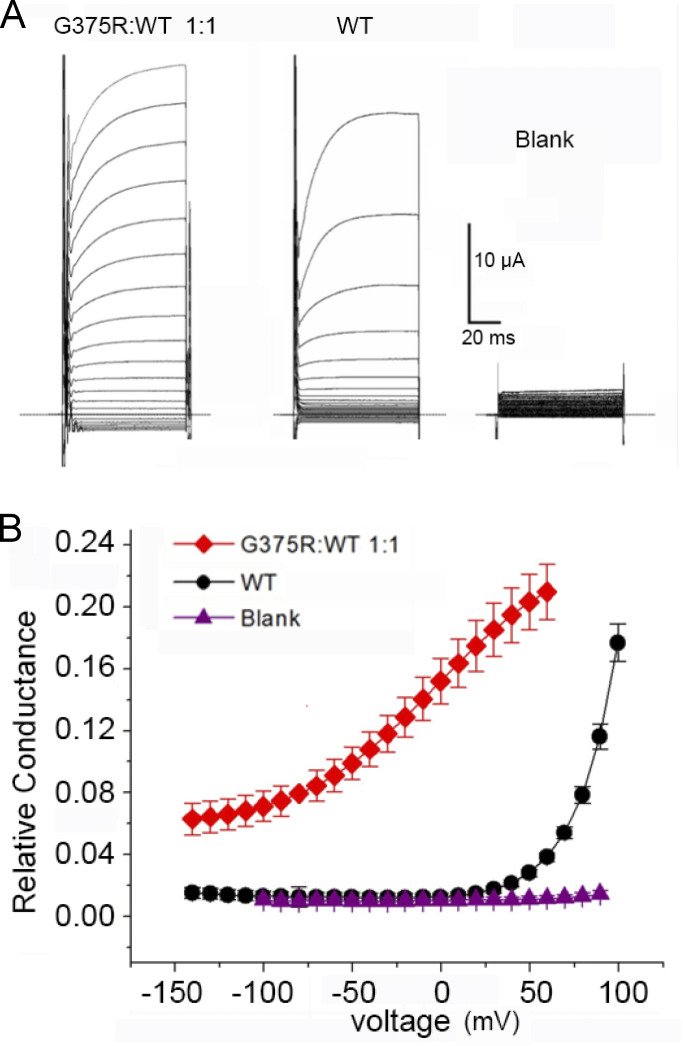
**BK channels expressed in oocytes following injection of a 1:1 mixture of G375R mutant and WT cRNA activate at greatly left-shifted negative potentials compared with BK channels expressed following injection of only WT cRNA. (A)** Whole-cell currents recorded from oocytes with the two-electrode voltage clamp for the indicated injections of cRNA. The whole cell currents were generated by holding the potential at −80 mV and then jumping to voltages ranging from −140 mV to +60 mV in 10 mV increments (1:1) or to +100 mV (WT and blank). **(B)** Plots of relative conductance versus the voltage of the steps following injection of a 1:1 mixture of mutant and WT cRNA (red diamonds); injection of WT cRNA alone (black circles); or injection of carrier only (purple triangles). The high conductance at negative potentials would act to drive the membrane potential to about −80 mV, the equilibrium potential of K^+^. Channel activation is left-shifted in the whole cell recordings in this figure compared to the essentially 0 Ca^2+^ macro patch recordings in Fig. 3 because the resting free Ca^2+^ in the oocytes of a few micromolar left shifts BK channel activation. The relative cord conductance was calculated from the plotted steady-state currents in Fig. S2 using the step potential minus the reversal potential for the voltage driving force. Mean ± SEM, *n* = 4 in each case.

**Figure S2.**
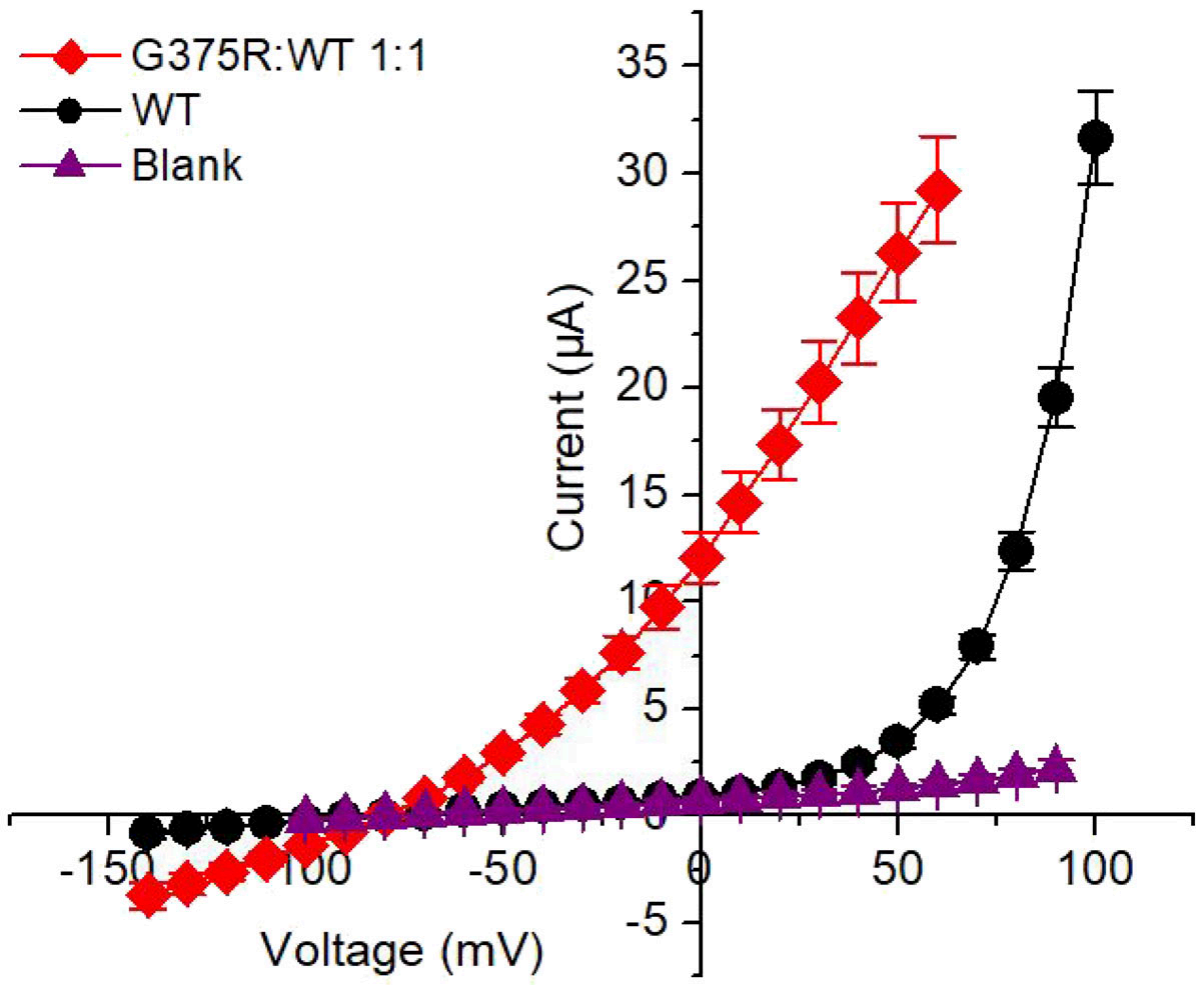
**BK currents in *Xenopus* oocytes following injection of a 1:1 mixture of G375R mutant and WT cRNA activate at greatly left-shifted negative voltages compared with BK currents following injection of only WT cRNA.** I-V plots for the indicated injections of cRNA and blank. Two-electrode whole-cell voltage clamp. These currents were used to calculate the relative conductance plotted in Fig. 2. Protocol details are in Fig. 2 legend. The amount of WT cRNA injected was 25 times greater than for the injection of the 1:1 mixture of G375R mutant and WT cRNA so that the initial deviation of WT currents from the baseline could be readily detected. Mean ± SEM, *n* = 4 in each case.

**Figure S3.**
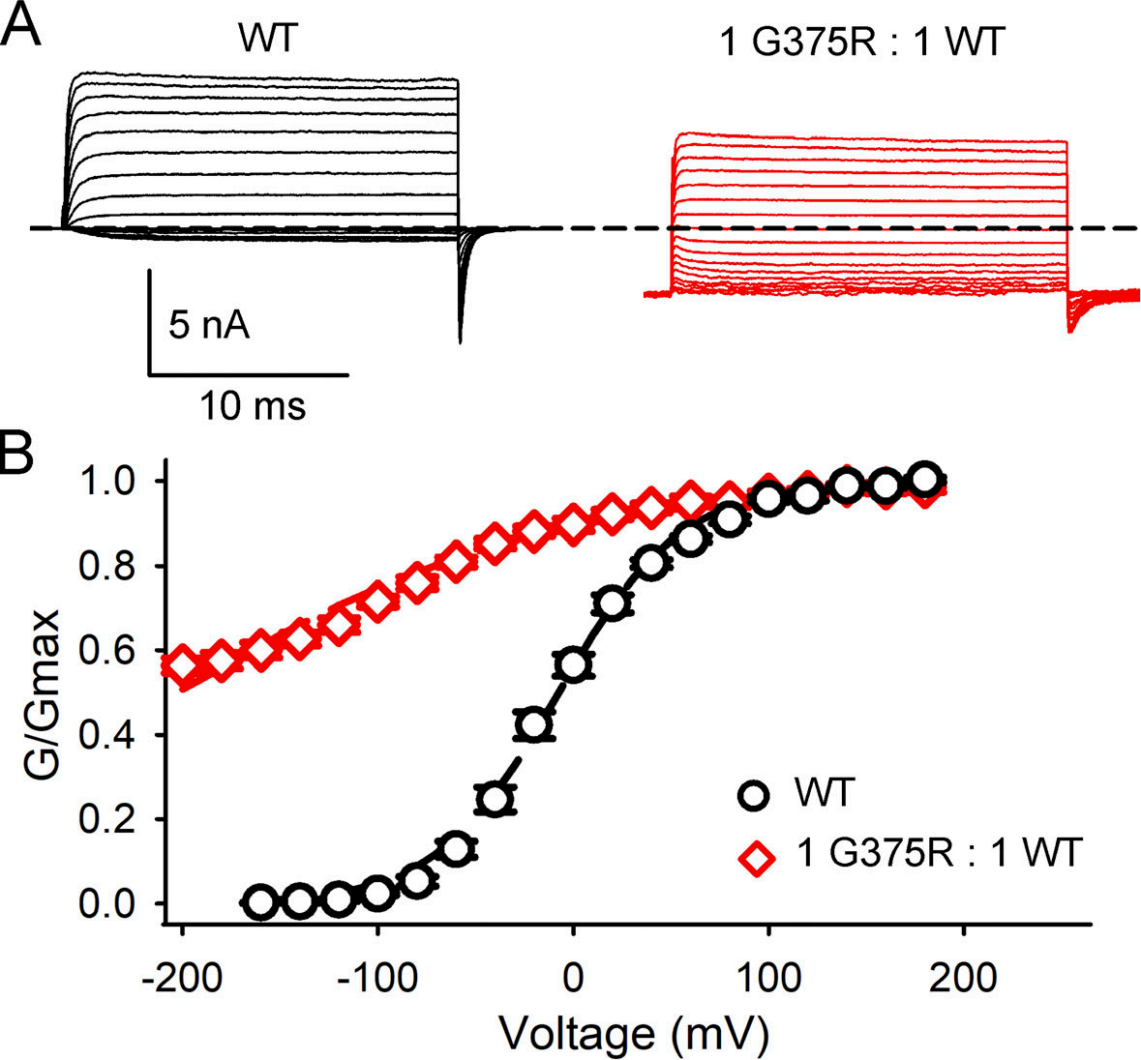
**The aberrant negative shift in *V***_**h**_ **for BK currents expressed following a 1:1 injection of G375R mutant and WT cRNA is also observed with 300 μM intracellular Ca**^**2+**^**. (A)** Currents recorded from inside-out macro patches of the membrane with 300 μM Ca^2+^ at the inner membrane surface. For WT channels, voltage pulses were from −160 to 180 mV with 20 mV steps from a holding potential of −160 mV. Conductance was measured from tail currents after stepping to −160 mV. For channels expressed following injection of a 1:1 mixture of G375R mutant and WT cRNA, voltage pulses were from −200 to 180 mV with 20 mV steps from a holding potential of −200 mV. Conductance was measured from tail currents after stepping to −200 mV. The dashed line indicates the level of 0 current. In the presence of 300 μM intracellular Ca^2+^, more than half of the channels expressed following the 1:1 injection of G375R mutant and WT cRNA remain open at −200 mV. The macrocurrents are the average response from many hundreds of BK channels in each macro patch. **(B)** G-V plots with 300 μM Ca^2+^ following injection of WT cRNA alone (black circles) or a 1:1 injection of mutant and WT cRNA (red diamonds). The mean *V*_h_ for WT currents was −7.2 ± 3.3 mV, *n* = 8, shifting to −205 ± 9.6 mV for BK currents expressed from a 1:1 mixture of mutant and WT cRNA, producing a left shift of −198 mV with 300 μM Ca^2+^ at the inner membrane surface, *n* = 6. This can be compared with a left shift of −120 mV with 0 Ca^2+^ (Fig. 3). Hence, the aberrant left shift in *V*_h_ induced by a 1:1 injection of mutant and WT cRNA occurs in the presence and absence of intracellular Ca^2+^.

**Figure S4.**
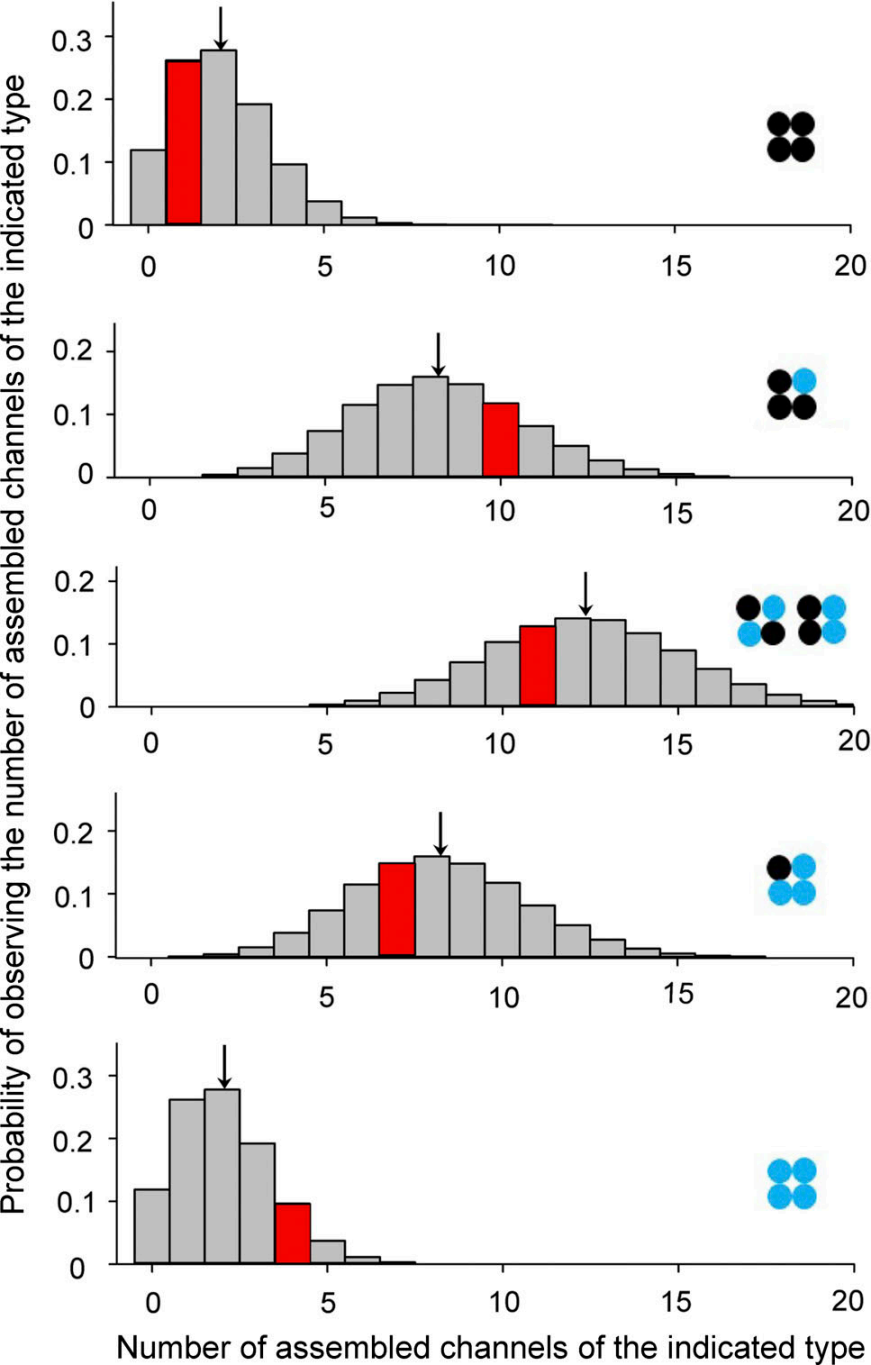
**The experimentally observed percentages of the five types of assembled channels expressed following a 1:1 injection of G375R mutant and WT cRNA did not differ significantly from the theoretical percentages calculated assuming random assembly of G375R mutant and WT subunits**. Simulation was used to determine if the percentages of observed and predicted channel types (Fig. 5 B and Fig. 1) were significantly different by examining whether the observed numbers of channels of each type in the experimental group of 33 assembled channels (Fig. 5 A) were significantly different from the expected numbers based on random assembly of subunits (this figure), as it is the numbers that determine the percentages. The expected numbers could not be calculated directly because of the random assembly of subunits but could be specified in terms of simulated discrete probability distributions, one for each of the five channel types (see Materials and methods). This figure plots the five discrete probability distributions, where the specific channel type (stoichiometry) for each distribution is indicated by a schematic, where black circles are WT subunits and blue circles are mutant subunits. Each one of the five discrete probability distributions plots the probability of observing 0, 1, 2, 3…33 assembled channels of the type indicated for that distribution in a group of 33 channels randomly assembled from mutant and WT subunits. The five discrete probability distributions were generated by first simulating 10^6^ groups of 33 randomly assembled channels (see Materials and methods and Fig. 1) and then binning the number of each type of assembled channel in each group of 33 channels into five frequency histograms, one for each channel type. The bins in each frequency histogram were then divided by 10^6^, the total counts in each frequency histogram, to normalize each frequency histograms to discrete probability distributions with an area of 1.0. To illustrate with some examples, the topmost discrete probability distribution, which gives the probabilities of observing different numbers of WT channels in a group of 33 assembled channels, indicates that the probability of observing 0 WT channels in a group of 33 randomly assembled channels is 0.119. The probability of observing 1 WT channel is 0.262, and the probability of observing 2 WT channels is 0.278, etc. Thus, 11.9, 26.2, and 27.8% of the groups of 33 assembled channels would have 0, 1, or 2 WT channels, respectively. The arrow at 2.06 WT channels indicates the mean number of WT channels per group of 33 assembled channels from the analysis of the 10^6^ groups. The expected mean number of WT channels per group of 33 channels can also be calculated directly from the percentages in Fig. 1, where 6.25% of the 33 assembled channels on average would be WT, giving a mean of 2.06 WT assembled channels per group of 33 assemble channels (0.0625 × 33 = 2.06). The discrete probability distributions for each of the four other types of assembled channels are also presented and can be interpreted in a similar manner. Our experimental observations of the number of assembled channels of each type in the experimental group of 33 assembled channels (Fig. 4 D; and Fig. 5, A and B) are indicated by the locations of the red histogram bars on each abscissa of the discrete probability distributions. The amplitude of the red bars gives the probability of observing the indicated number of channels of that type assuming a random assembly of subunits. For example, the probability for our observation of 1 WT channel in the experimental group of 33 assembled channels was 0.262, and the probabilities for our observations of 10, 11, 7, and 4 assembled channels for the other indicated types of assembled channels in the experimental group of 33 assembled channels were 0.118, 0.128, 0.147, and 0.0961, respectively. All of these probabilities for the observed numbers of assembled channels of each type were >0.05, and none of the observed numbers of assembled channel types fell within the 0.05 summed cumulative probability of the left and right tails of the discrete probability distributions, indicating that the experimental observations for the number of the five types of assembled channels were not significantly different (Colquhoun, 1971) from the theoretical predictions based on an assumption of random subunit assembly. A lack of significant difference does not exclude that there may be underlying differences between experimental and theoretical predictions, which could be revealed by larger sample sizes.

Fig. S1 presents ribbon structures of selected parts of BK channels to show the location and size of an arginine sidechain compared with a glycine sidechain for the G375R mutation. Fig. S2 shows the currents recorded from *Xenopus* oocytes that were used to calculate the relative conductances plotted in Fig. 2. Fig. S3 presents G-V curves for macropatch data obtained in the presence of 300 μM Ca^2+^ to show that the G375R-induced negative shift also occurs in the presence of 300 μM Ca^2+^. Fig. S4 presents a statistical test showing that the observed percentages of the five different types of channels expressed when mimicking a heterozygous mutation were not significantly different from the theoretical percentages calculated for an assumption of random assembly of mutant and WT subunits.

## Results

### G375R subunits left shift BK channel activation

To examine what effect a de novo G375R mutation of the pore-forming subunit of BK channels would have on channel function when coexpressed with WT subunits (Fig. 1), we first compared the whole-cell macroscopic currents flowing through large numbers of WT BK channels expressed following injection of WT cRNA into *Xenopus* oocytes with those currents expressed following injection of a 1:1 mixture of mutant and WT cRNA to mimic a heterozygous mutation. The injection of a blank without cRNA served as a control. Voltages were stepped from a holding potential of −80 mV to more negative and more positive voltages to reveal the voltage dependent activation of the expressed currents (Fig. 2 A and Fig. S2). Voltage steps to +50 mV were required to appreciably activate WT BK currents generated from the injection of WT cRNA. In contrast, following a 1:1 injection of G375R mutant and WT cRNA, the expressed BK currents were hyperactive, being already active at −140 mV, with depolarization further increasing the response (Fig. 2 B and Fig. S2). The aberrant BK channels from the 1:1 injection that are responsible for these hyperactive currents could be either homotetrameric mutant channels, hybrid channels, or both (Fig. 1).

The negative shift in activation for currents expressed from the 1:1 injection was so pronounced that large numbers of aberrant BK channels were open at voltages near the resting membrane potential of −60 mV. If aberrant currents expressed similarly in neurons, the flux of K^+^ through opened BK channels would act to drive the membrane potential toward the equilibrium potential for K^+^, opposing depolarization of the cell and facilitating repolarization, both of which could interfere with normal neuronal function. Thus, at the level of whole-cell recording, the heterozygous G375R mutation gave a pronounced GOF phenotype because much less depolarization was required to activate BK currents. Some of the disease phenotypes associated with this mutation might be due to such GOF behavior. In contrast, Liang et al. (2019) reported a loss-of-function (LOF) for G375R because they observed no BK currents from HEK293T cells following transfection with G375R cDNA. Their conclusion of a functional LOF vs. our conclusion of a functional GOF will be considered in detail in a later section.

**Figure 3.**
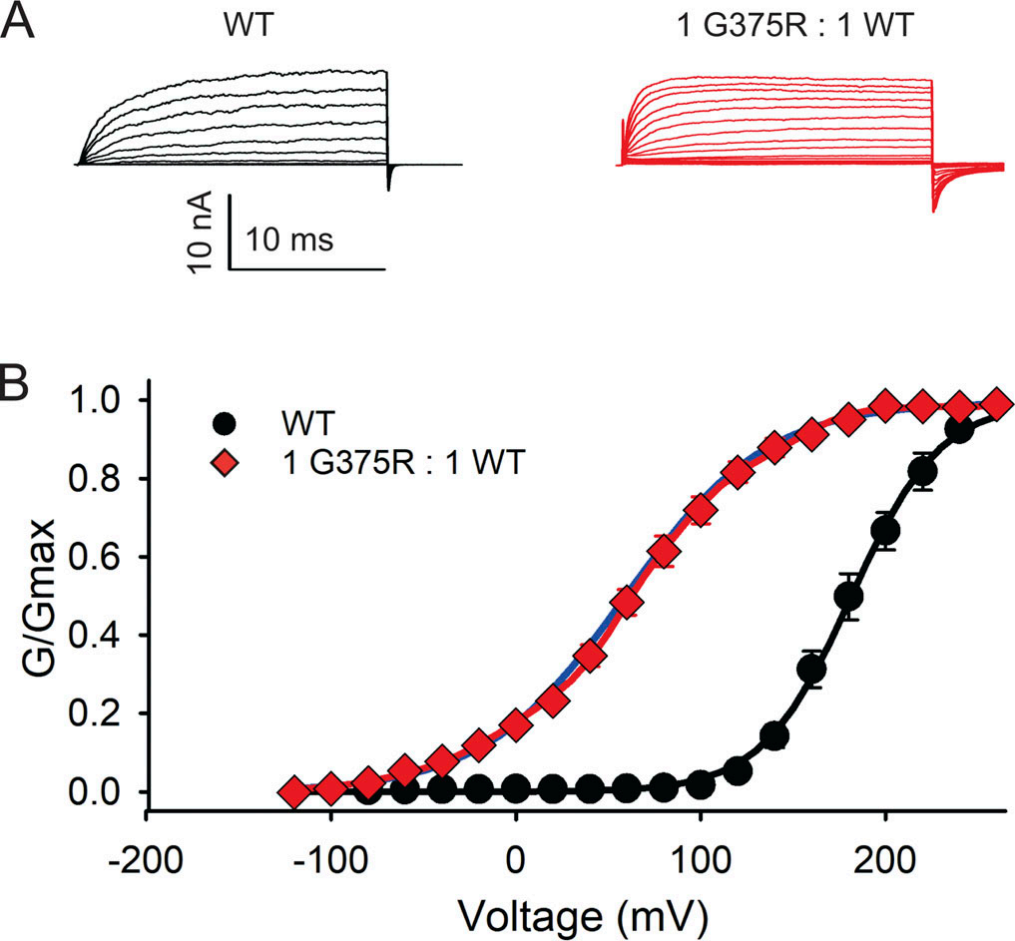
**Quantifying the mean negative shift in *V***_**h**_ **following injection of a 1:1 mixture of G375R mutant and WT cRNA when compared to injection of only WT cRNA.** Data are for essentially 0 Ca^2+^ at the intracellular side of the membrane for Figs. 3, 4, 5, and 6. **(A)** Currents recorded from inside-out macro patches of membrane excised from oocytes. For WT channels following injection of only WT cRNA, the holding potential was −80 mV and voltage pulses were from −80 to 240 mV with 20 mV steps followed by a step back to −80 mV to elicit tail currents for G/G_max_ measurements. For channels expressed following injection of a 1:1 mixture of G375R mutant and WT cRNA, the holding potential was −160 mV and voltage pulses were from −120 to 240 mV with 20 mV steps followed by a step back to −120 mV to elicit tail current for measurement. The currents are the mean response from the many hundreds to thousands of BK channels in each excised macropatch of membrane. **(B)** G-V plots following the injection of WT cRNA alone (black circles, *n* = 6; mean ± SEM), or 1:1 injection of mutant and WT cRNA (red diamonds, *n* = 12; mean ± SEM). The black line through the WT data is a single Boltzmann function with *V*_h_ = 181.3 mV and slope factor *b* = 24.04 mV/e-fold change in Po. The blue line through the 1:1 data (red diamonds) is a single Boltzmann function with *V*_h_ = 59.04 mV and *b* = 38.06 mV/e-fold change. The red line through the 1:1 data is the sum of five Boltzmann functions with (1) *V*_h_ = −75.7 mV, *b* = 21.6 mV/e-fold change, fractional area = 0.0547; (2) 1.5, 21.0, 0.236; (3) 54.6, 13.8, 0.316; (4) 96.6, 14.4, 0.244; (5) 155, 14.0, 0.139. These parameters for the five summed Boltzmann functions that describe the 1:1 data are generally consistent with the *V*_h_ values and percentages for the apparent clusters of assembled channels (Fig. 5, A and B) and theoretical percentages in Fig. 1.

To quantify the average negative voltage shift in activation for the BK currents following injection of a 1:1 mixture of G375R and WT cRNA when compared with WT currents following injection of only WT cRNA, we recorded currents from macropatches of the membrane which were excised from oocytes after channel expression. This allowed the composition of the solution at the inner membrane surface to be controlled, which was not the case for the whole-cell recordings in Fig. 2. For a solution at the intracellular membrane surface containing <0.01 μM Ca^2+^ (0 Ca^2+^), WT BK currents did not activate appreciably until the membrane potential exceeded 100 mV (black circles), whereas BK currents from the 1:1 injection of G375R mutant and WT cRNA started to activate at negative voltages of −80 mV (Fig. 3 B). The mean *V*_h_ was 182 ± 5.4 mV (*n* = 6) for WT BK currents and 61.5 ± 16 mV (*n* = 12) for BK currents from the 1:1 injection, giving a mean left shift of −120 mV in voltage activation for the currents following a 1:1 injection when compared with WT (P < 0.0001). The 1:1 injection also decreased the voltage sensitivity of activation compared with WT with a slope of 38.5 ± 1.8 (*n* = 8) mV per e-fold change compared with 23.2 ± 1.36 mV (*n* = 6) for WT currents (P < 0.0001).

The G375R-induced negative shift in voltage activation and reduced slope were even greater in the presence of 300 μM Ca^2+^ (Fig. S3). Thus, the large negative shift in voltage activation observed for whole-cell recordings following a 1:1 injection of mutant and WT cRNA (Figs. 2 and S2) was also observed for macropatch recordings under conditions in which the intracellular solution and Ca^2+^ were controlled (Figs. 3 and S3).

Whole-cell and macropatch recordings are useful to show the average response of many hundreds to thousands of BK channels, but they provide limited information about the properties of the underlying channels, unless all channels are identical, which is unlikely to be the case for a heterozygous mutation, as shown in Fig. 1. To investigate the channels underlying the negative shifts in BK currents in Figs. 2 and 3 for a 1:1 injection of mutant and WT cRNA to mimic a mutation heterozygous with the WT allele, we first considered the possible channel types and percentages of expression that might be expected for a heterozygous mutation. We then recorded from individual channels expressed following a 1:1 injection of mutant and WT cRNA to see if the expectations were met.

### BK channels of five different potential stoichiometries and six different subunit arrangements could theoretically be expressed for a mutation heterozygous with WT

WT and mutant subunits arising from a heterozygous mutation could potentially assemble into tetrameric BK channels with five different stoichiometries and six different subunit arrangements, each with potentially different properties (Fig. 1; MacKinnon, 1991; Blaine and Ribera, 1998; Niu and Magleby, 2002; Bergendahl et al., 2019; Backwell and Marsh, 2022). If it is assumed that there is equal production of mutant and WT subunits, that subunits assemble randomly to form tetrameric channels, and that each assembled channel has the same probability of reaching the surface membrane, then the channels expressed for a heterozygous mutation would consist of 6% WT channels, 25% comprised of one mutant and three WT subunits, 38% comprised of two mutant and two WT subunits, 25% comprised of three mutant and one WT subunit, and 6% homotetrameric mutant channels (Fig. 1). On this basis, 94%, of the expressed channels could be pathogenic, comprised of 88% hybrid (heterotetrameric) channels of mixed subunits and 6% homotetrameric mutant channels, with only 6% WT channels (Fig. 1; see unrounded percentages in the figure legend).

### Assembled channels

The term assembled channels will be used to refer to those BK channels that are assembled and expressed following injection of a 1:1 mixture of G375R mutant and WT cRNA into *Xenopus* oocytes to mimic a heterozygous mutation. Assembled channels could theoretically include five types of channels with different stoichiometries and six types if subunit arrangement is considered (Fig. 1). An individual assembled channel could then be any one of the six channel types. It is the combined activity of the six potential types of assembled channels that would determine the BK currents for a heterozygous mutation.

### Assembled channels display an abnormally wide range of *V*_h_

**Figure 4.**
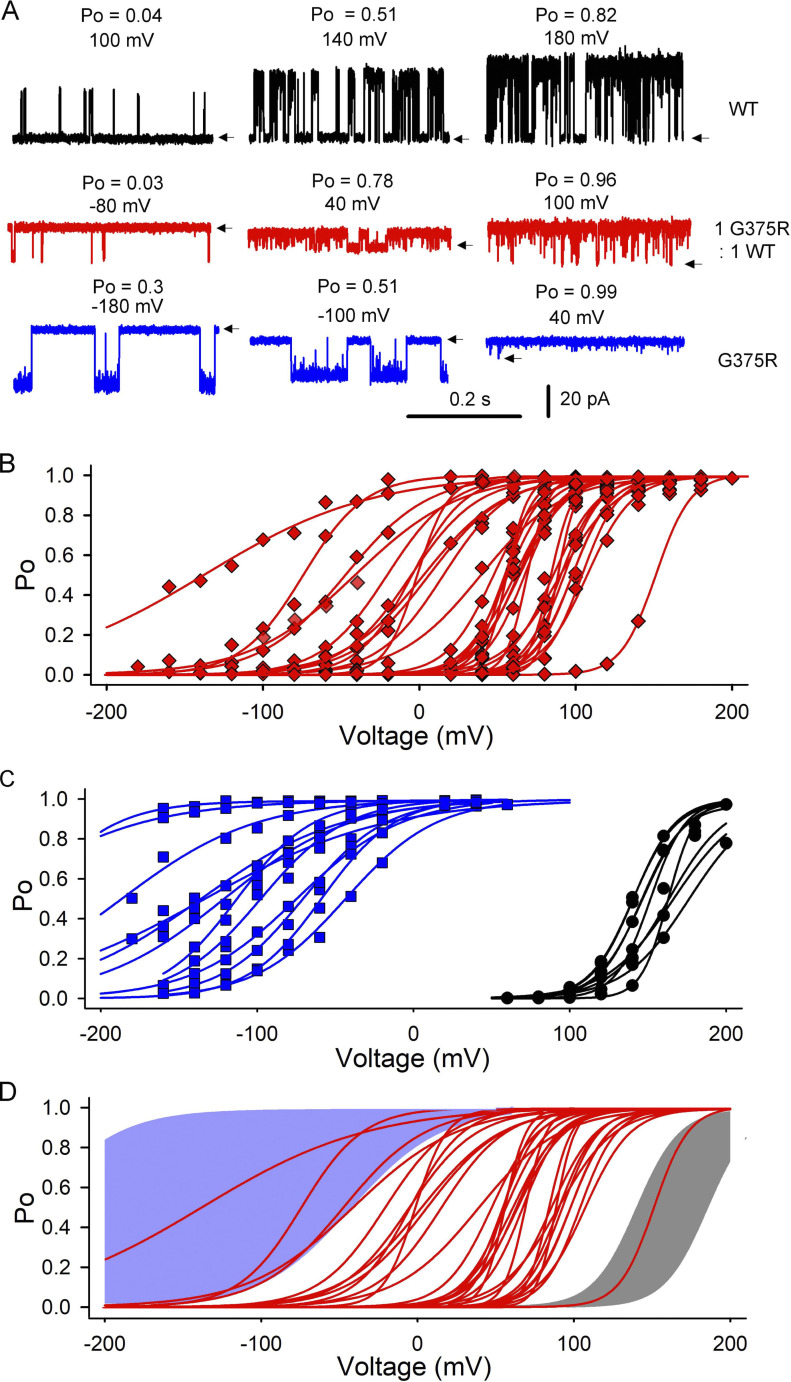
**Identifying the types of assembled BK channels expressed following a 1:1 injection of G375R mutant and WT cRNA. (A)** Representative single-channel recordings from three different single BK channels: WT channel (top row) following injection of WT cRNA; an assembled channel following 1:1 injection of G375R mutant and WT cRNA, (second row); and a G375R homotetrameric mutant channel following injection of G375R mutant cRNA (third row). Recordings are shown at three different voltages for each channel type. Depolarization activates each channel type with a markedly different *V*_h_ of 140 mV for the WT channel, 6 mV for the assembled channel, and −100 mV for the G375R homotetrameric mutant channel. Arrows indicate closed channel current levels. **(B)** Plots of Po-V for 33 individual assembled channels following a 1:1 injection of mutant and WT cRNA (red diamonds with Boltzmann fits). **(C)** Plots of Po-V for 12 single G375R homotetrameric mutant channels following injection of G375R mutant cRNA (blue squares with Boltzmann fits), and for nine WT channels following injection of WT cRNA (black circles and Boltzmann fits). **(D)** Identifying the types of assembled channels. Blue and gray areas indicate the range of observed *V*_h_ values for G375R homotetrameric mutant channels and WT channels, respectively, from C. One of the assembled channels (rightmost red Po-V curve) overlaps with the WT channels (gray shading), indicating that this assembled channel is likely a WT channel with four WT subunits. Four of the assembled channels (four leftmost red Po-V curves) overlap with the G375R homotetrameric mutant channels (blue shading) suggesting that these four assembled channels are G375R homotetrameric mutant channels assembled from four mutant subunits. The remaining 28 assembled channels would be hybrid channels with mixed subunits (Fig. 1), as their *V*_h_ values do not overlap with those of either homotetrameric mutant or WT channels.

To explore if multiple channel types contribute to the negative shift in *V*_h_ resulting from a G375R BK subunit mutation heterozygous with WT (Figs. 2, 3, S2, and S3), we used the unique ability of the patch clamp technique (Hamill et al., 1981; Niu and Magleby, 2002; Gonzalez-Perez et al., 2015) to isolate and record from individual assembled channels expressed following a 1:1 injection of mutant and WT cRNA into *Xenopus* oocytes. For each of the 33 individual assembled channels studied, single-channel currents were recorded over a range of voltages (Fig. 4 A) to obtain plots of Po-V (Fig. 4 B, red curves). The Po-V plots for the 33 individual assembled channels had a *V*_h_ that ranged from −152 mV to +151 mV, for a span of 303 mV (Fig. 4 B, red curves). This very wide range in *V*_h_ supports the idea that there are multiple types of assembled channels with different properties arising from different subunit compositions (Fig. 1).

### Assembled channels include WT, homotetrameric mutant, and hybrid channels

To identify the types of BK channels expressed following a 1:1 injection of mutant and WT cRNA, the Po-V curves of the 33 individual assembled channels in Fig. 4 B were compared with Po-V curves obtained from individual WT and homotetrameric mutant channels. The WT and homotetrameric mutant channels were obtained by single-channel recording after injection of only WT cRNA or only mutant cRNA, respectively. The WT channels had *V*_h_ values that spanned a narrow range from +140 to +176 mV (Fig. 4 C, black Po-V curves), and the homotetrameric mutant channels had *V*_h_ values that ranged from −272 mV to −38 mV (Fig. 4 C, blue Po-V curves). The homotetrameric mutant channels had a surprisingly wide range of *V*_h_ for channels of the presumed identical composition of four mutant subunits, but all were in a far more negative voltage range than WT channels. The reason for such a wide voltage range in *V*_h_ for the homotetrameric mutant channels is not known, but perhaps channels with four mutant subunits can assume different conformations with markedly different activation properties, depending on mutant side-chain orientation (Fig. S1).

To facilitate the identification of the types of assembled channels, the Po-V curves of the individual 33 assembled channels from Fig. 4 B were overlaid on plots of the *V*_h_ ranges of the Po-V control curves for known WT (gray shading) and homotetrameric mutant (blue shading) channels in Fig. 4 D. One of the 33 assembled channels had a Po-V curve that overlapped with the known WT channel controls (Fig. 4 D), suggesting that this assembled channel was WT with four WT subunits. 4 of the 33 assembled channels had Po-V curves that overlapped with the known homotetrameric mutant channel controls (Fig. 4 D), suggesting that these four assembled channels were homotetrameric mutants comprised of four mutant subunits. The remaining 28 assembled channels had Po-V curves that did not overlap with the known WT or homotetrameric mutant channel controls (Fig. 4 D) but fell in between, suggesting that these 28 assembled channels were all hybrid channels comprised of a mix of mutant and WT subunits (Fig. 1). In this study, assembled channels with *V*_h_ values in the ranges of WT, hybrid, and homotetrameric mutant channels will be referred to as WT, hybrid, and homotetrameric mutant channels, respectively, with the understanding that these classifications are based on *V*_h_ values.

For the sampled group of 33 assembled channels, ∼3% (1/33) were consistent with WT, ∼85% (28/33) with hybrid, and ∼12% (4/33) with homotetrameric mutant (Fig. 4, B–D). Fig. 1 predicts ∼6% WT, ∼88% hybrid, and ∼6% homotetrameric mutant channels. Thus, for the G375R mutation heterozygous with WT, both experimental and theoretical considerations suggest that most (85–88%) of the expressed assembled channels will be hybrid, with much smaller fractions of homotetrameric mutant and WT channels. All hybrid and homotetrameric mutant channels displayed negative shifts in activation compared with WT, with the greatest negative shifts for the homotetrameric mutant channels. Consequently, both theoretical and experimental considerations suggest that 94–97% of the BK channels arising from a G375R mutation heterozygous with WT would display aberrant negative shifts in activation, even though only 50% of the subunits synthesized in a cell would be mutant.

### Three functional types of hybrid assembled channels

**Figure 5.**
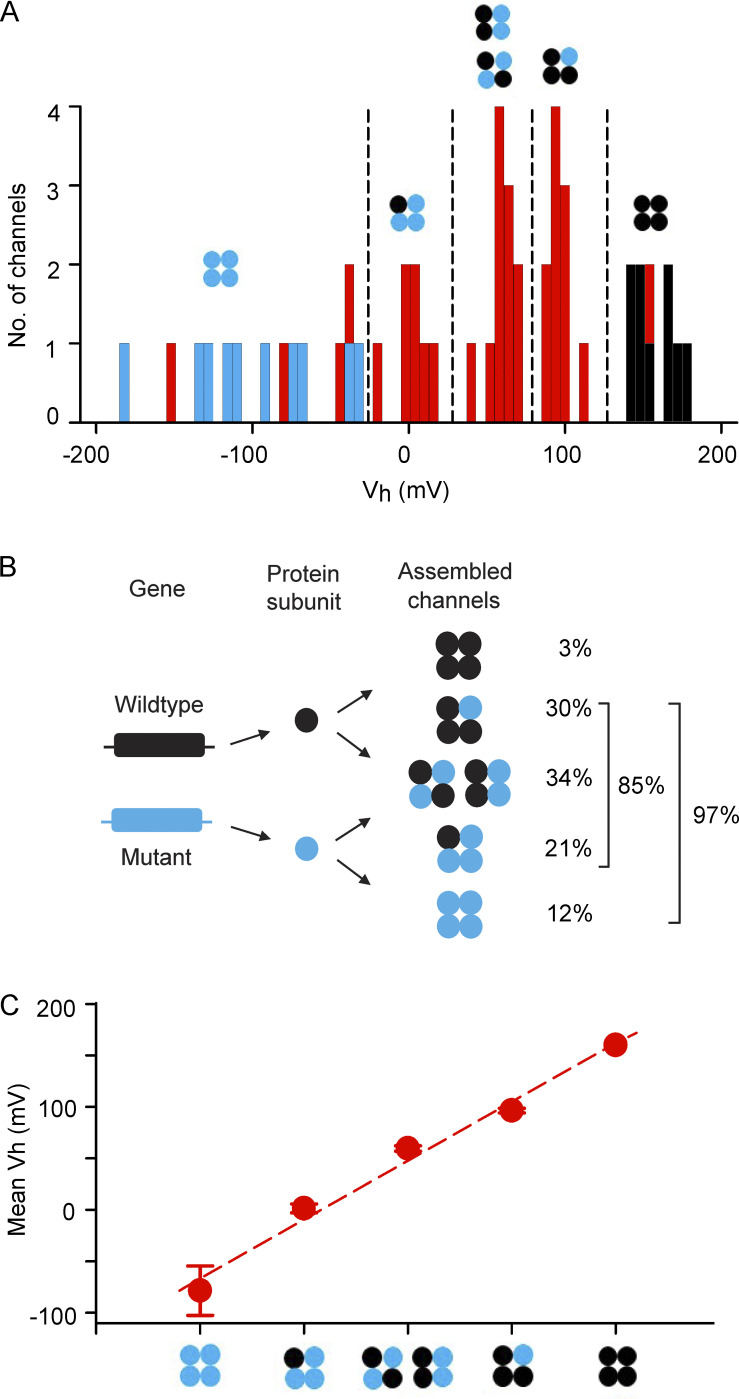
**Five different types of functional BK channels are observed following injection of a 1:1 mixture of mutant and WT cRNA into *Xenopus* oocytes. (A)** Histograms of *V*_h_ values of single-channels from Fig. 4. WT channels expressed after injection of only WT cRNA are indicated by black bars, and G375R homotetrameric mutant channels expressed after injection of only G375R cRNA are indicated by blue bars. The 33 assembled channels expressed after a 1:1 injection of mutant and WT cRNA are indicated by red bars. The one assembled channel in the cluster of black bars is consistent with a channel comprised of four WT subunits. The four assembled channels within the blue bars are consistent with channels comprised of four mutant subunits. The 28 assembled channels with *V*_h_ values falling between those of the blue bars and black bars are consistent with hybrid (heterotetrameric) channels assembled from mixed subunits. The 28 hybrid channels appear to cluster into three groups with mean ± SEM *V*_h_ values of 1.4 ± 4.3 mV for the 7 channels in the left hybrid group, 59.6 ± 2.6 mV for the 11 channels in the middle hybrid group, and 96.4 ± 2.2 mV for the 10 channels in the right hybrid group. All *V*_h_ comparisons of the three hybrid groups were significantly different, P < 0.0001, two-tailed *t* test. The single assembled channel that fell within the WT (black) group had a *V*_h_ of 151.3 mV, which was significantly different from the 96.4 mV hybrid group, P < 0.0001, one sample two-tailed *t* test. The group of four hybrid channels that fell within the homotetrameric (blue) group had a *V*_h_ of −75.0 ± 22.0 mV which was significantly different from the 1.4 mV hybrid group, P = 0.0019, two-tailed *t* test. Hypothesized subunit compositions of the three apparent clusters of hybrid channels are depicted by schematics, where black circles indicate WT subunits and blue circles indicate G375R mutant subunits. Subunit structures for the homotetrameric mutant and WT channels are also indicated. **(B)** The experimentally observed percentages for each of the five types of 33 assembled channels are listed for comparison to the theoretical percentages from Fig. 1. **(C)** *V*_h_ for the five types of assembled channels is approximated by a linear incremental model where each subunit contributes an increment of *V*_h_, $V_{h\left(NM\right)}=\left(N_{M}\right)\left(-16.7mV\right)+\left(4-N_{M}\right)\left(40.5mV\right)\text{,}$ where *N*_M_ is the number of G375R mutant subunits per channel, 4-*N*_M_ is the number of WT subunits per channel, *V*_h(*N*M)_ is *V*_h_ as a function of *N*_M_, and −16.7 and 40.5 mV are the increments of *V*_h_ added by each mutant and WT subunit, respectively. Filled red circles are the experimentally observed mean ± SEM values of *V*_h_ for the five types of assembled channels from A, and the dashed line indicates the predicted values. Data are for 0 intracellular Ca^2+^. Histograms of single-channel kinetic parameters have been used previously to investigate the number of β2 regulatory subunits per BK channel (Wang et al., 2002).

Analysis in Fig. 4 suggested that assembled channels comprised of 85% hybrid channels, 3% WT, and 12% homotetrameric mutant (Fig. 4). The 85% hybrid channels themselves could consist of three or four different functional types based on both subunit composition and arrangement (Fig. 1). If the functional properties of hybrid channels depend only on subunit stoichiometry, then three functional types would be expected, for one, two, or three mutant subunits replacing an equal number of WT subunits in the heterotetrameric hybrid channels. In addition, if hybrid channels comprising of two mutants and two WT subunits displayed different functional properties for adjacent or diagonal subunit arrangement, then four types of hybrid channels might be expected (Fig. 1). To assess the number of functional types of hybrid channels, a histogram of the *V*_h_ values of the 33 assembled channels from Fig. 4 B was plotted in Fig. 5 A as red bars. Histograms of known WT channel controls (black bars) and homotetrameric mutant channel controls (blue bars) from Fig. 4 C are also plotted. The subunit compositions of the homotetrameric mutant and WT channel controls are known and placed above these two types of channels in Fig. 5 A. As expected from Fig. 4 D, four of the assembled channels had *V*_h_ values that overlapped with those of homotetrameric mutant channels, suggesting that they were homotetrameric mutant channels; one assembled channel overlapped with WT, suggesting that it was a WT channel; and the remaining 28 assembled channels had *V*_h_ values falling in between those of homotetrameric mutant and WT channels, suggesting they were hybrid channels (Fig. 1) whose properties were determined by mixtures of mutant and WT subunits.

The *V*_h_ values of the 28 hybrid channels fell into three apparent clusters with mean values of about 1.4, 59, and 96 mV (Fig. 5 A). Three clusters of *V*_h_ for hybrid channels would be expected from Fig. 1 if each mutant subunit replacing a WT subunit added an increment of a negative shift in *V*_h_, independent of subunit arrangement. Accordingly, as a working hypothesis, the different subunit combinations for hybrid channels from Fig. 1 have been placed above the three clusters of hybrid channels in Fig. 5 A to obtain a stepwise negative shift in *V*_h_ for each additional mutant subunit replacing a WT subunit. Since only three clusters of hybrid channels were observed, instead of four, it was assumed that the hybrid channels with two mutant and two WT subunits in either adjacent or diagonal subunit arrangement had similar *V*_h_ values so that they contributed to the same cluster. Small differences in *V*_h_ could be obscured by variability in *V*_h_ inherent in single-channel data. Fig. 5 A then suggests five different functional types of assembled channels: homotetrameric mutant, three types of hybrid channels, and WT channels, as indicated.

The percentages of expression of the five functional types of assembled channels were then calculated from the number of assembled channels in each cluster in Fig. 5 A and presented in Fig. 5 B. The results are consistent with 3% WT channels, 12% homotetrameric mutant channels, and 85% hybrid channels, where 30% of the hybrid channels had one mutant and three WT subunits, 34% had two mutant and two WT subunits, and 21% had three mutant and one WT subunits. These percentages can be compared with the theoretical values in Fig. 1 calculated for equal production, and a random assembly of subunits where 6% were WT channels, 6% were homotetrameric mutant channels, and 88% were hybrid channels, where 25% of the hybrid channels had one mutant and three WT subunits, 38% had two mutant and two WT channels, and 25% had three mutant and one WT channels. Simulation of 1 million groups of 33 assembled channels, assuming equal production of mutant and WT subunits followed by random assembly into tetrameric channels, indicated that the experimentally observed percentages in Fig. 5 B were not significantly different (Fig. S4) from the theoretical predictions in Fig. 1. A lack of significance does not exclude the possibility that limited amounts of preferential production and assembly of mutant and WT subunits also contributed to the differences between observed and predicted percentages, in addition to the large variability arising from the random assembly of subunits that are characterized in Fig. S4.

Whereas the observation of three apparent clusters of *V*_h_ values for the hybrid assembled channels (Fig. 5) is consistent with theoretical predictions (Fig. 1), peaks can occur by chance alone in histograms of binned data of limited sample size (Miller et al., 1978). Consequently, additional experiments would be needed to determine whether *V*_h_ values for hybrid channels can consistently be resolved into three peaks, but an observation of such distinct peaks is not required for support of Fig. 1, as variability in *V*_h_ among channels of the same type (McManus and Magleby, 1991) might be sufficient to obscure distinct peaks.

### Linear incremental model for the contributions of mutant and WT subunits to *V*_h_

WT BK channels comprised of four WT subunits had a mean *V*_h_ of about 160 mV, and homotetrameric mutant BK channels comprised of four mutant subunits had a mean *V*_h_ of about −80 mV (Fig. 4 and Fig. 5 A). As all four subunits contribute to the gating of BK channels (Rothberg and Magleby, 2000; Horrigan and Aldrich, 2002; Niu and Magleby, 2002), these observations suggest that each WT subunit contributes an increment of a positive shift to *V*_h_ and that each mutant subunit contributes an increment of negative shift. In support of this hypothesis, a linear incremental model in which each WT subunit in a channel added +40.5 mV to *V*_h_ and each mutant subunit added −16.7 mV provided an approximate description of the observed *V*_h_’s for the five types of assembled channels (Fig. 5 C dashed line; model in the figure legend). The net effect of replacing a WT subunit with a mutant subunit was a −57.2 mV shift in *V*_h_ to account for the loss of the positive shift contributed by the removed WT subunit and the addition of the negative shift contributed by the added mutant subunit.

The mechanism by which each subunit with a G375R mutation provides a net −57.2 mV shift in *V*_h_ is not known, but is unlikely to involve the addition of positive charge by arginine because the G375D mutation (G310D in Chen et al., 2014) which adds negative charge also gives a negative shift in *V*_h_, but of smaller magnitude. That the left shift in *V*_h_ increases with side chain volume, where arginine > aspartate > glycine suggests that larger side chain volumes at the hinge position of G375 may progressively decrease the energy barrier for opening.

### Dual action of G375R subunits on *V*_h_ and single channel conductance *g*

**Figure 6.**
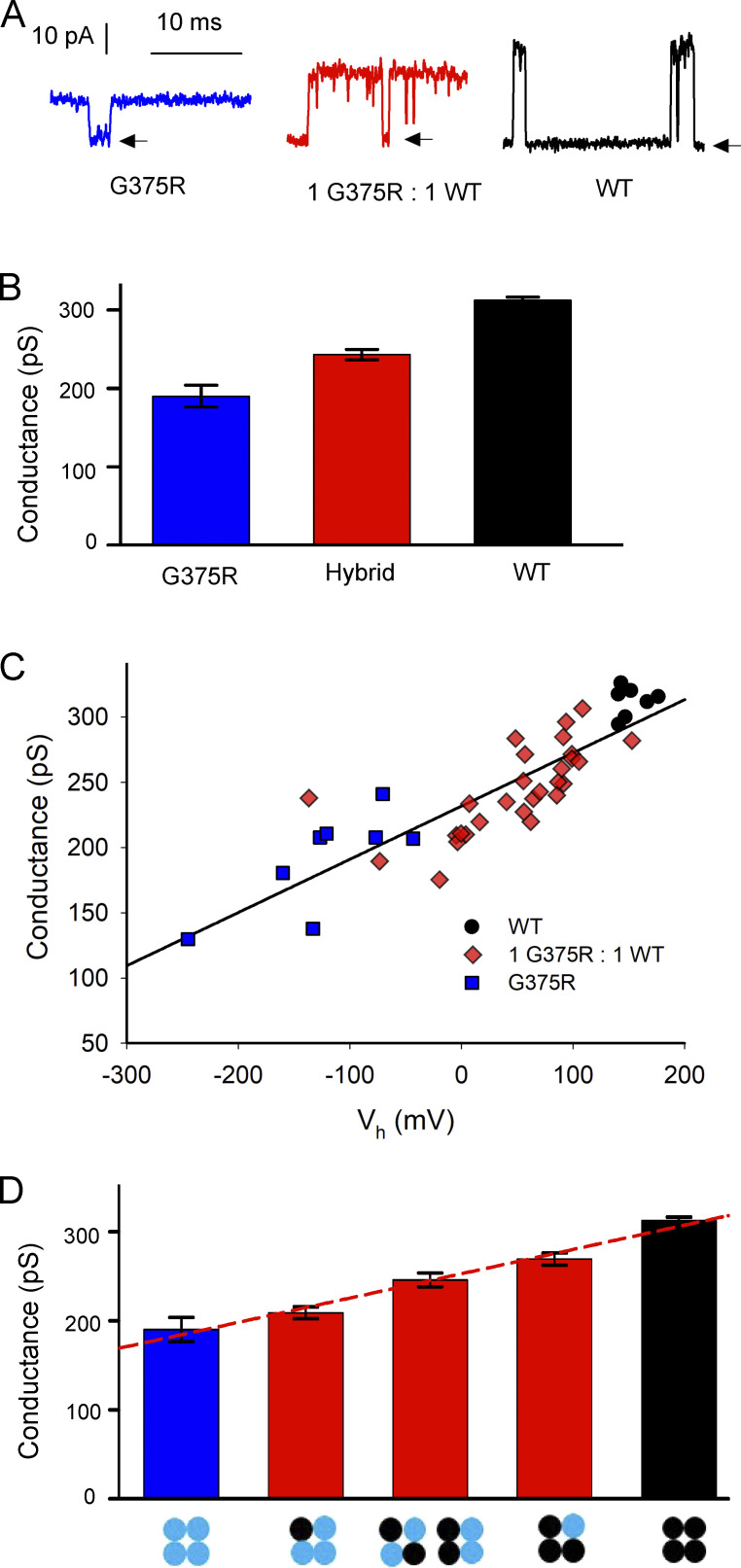
**Each replacement of a WT subunit with a G375R mutant subunit in a BK channel acts to both left shift *V***_**h**_ **to more negative potentials and decrease single channel conductance. (A)** Single-channel currents were recorded from single channels at +100 mV after injecting the indicated cRNA. **(B)** Single-channel conductance *g* at +100 mV decreases as the number of mutant subunits increases. Mean ± SEM single-channel conductance for WT channels was 312 ± 4 pS (*n* = 7), decreasing to 245 ± 6 pS for hybrid channels (*n* = 25, P < 0.0001), and further decreasing to 190 ± 14 pS for G375R homotetrameric mutant channels (*n* = 8, P = 0.0003). Mean ± SEM. WT and G375R homotetrameric mutant channels were those channels expressed after injecting only WT cRNA or only G375R mutant cRNA, respectively. Hybrid channels were identified as in Fig. 4 D. **(C)** Plot of single channel conductance at +100 mV vs. *V*_h_ for BK channels expressed following injection of the indicated cRNA. The linear regression line plots single channel conductance *g* vs. *V*_h_, where *g* = *g*(0) + *aV*_h_, where *g*(0) = 231.9 ± 3.9 pA, and *a* = 0.408 ± 0.0375 pS/mV (P < 0.0001 for slope significantly different from 0. R = 0.86). **(D)** Single-channel conductance for the five types of assembled channels is approximated by a linear incremental model, where each subunit contributes an increment of conductance, $g_{\left(NM\right)}=\left(N_{M}\right)\left(46.1pS\right)+\left(4-N_{M}\right)\left(76.5pS\right)\text{,}$ where *N*_M_ is the number of G375R mutant subunits per channel, 4-*N*_M_ is the number of WT subunits per channel, *g*_(*N*M)_ is single channel conductance as a function of *N*_M_, and 46.1 pS and 76.5 pS are the increments of *g* added by each mutant and WT subunit, respectively. The red dashed line indicates the predicted values of *g*. The homotetrameric mutant (blue) and WT (black) conductance bars are from B. The three red hybrid conductance bars plot the mean ± SEM of *g* for the three groups of hybrid channels identified in Fig. 5 A, where *g* measurements were available at +100 mV, giving: 209.0 ± 6.7 pS, *n* = 7; 246.0 ± 7.7 pS, *n* = 8; and 269 ± 2.6 pS, *n* = 10. For the four possible pairs of adjacent single-channel conductance bars in D, P values were calculated for *g* of the right conductance bar in each pair being significantly different than *g* for the left, and were 0.26, 0.0034, 0.0064, 0.0002, respectively; two-tailed *t* tests.

In addition to the negative shift in activation induced by replacing WT subunits with G375R mutant subunits (Figs. 2, 3, 4, and 5), replacing WT subunits with mutant subunits also decreased single-channel conductance (Fig. 6, A and B). The mean single-channel conductance of WT channels was 312 ± 4 pS. This decreased to 245 ± 6 pS for hybrid channels and further decreased to 190 ± 14 pS for homotetrameric mutant channels. These decreases were significant (Fig. 6 legend). Hence, single-channel conductance decreased as mutant subunits replaced WT subunits. The decreased single-channel conductance may arise from the larger volume and positive charge of the mutant arginine side chains replacing the single hydrogen atom of the glycine side chains on one or more of the S6 segments lining the conductance pathway of the BK channel (Fig. S1). The added volume of the sidechains could decrease the volume of the inner vestibule and the added positive charge may act to repel K^+^ from the inner cavity. Both actions can reduce single-channel conductance in BK channels (Brelidze et al., 2003; Geng et al., 2011; Nimigean et al., 2003).

To examine the relationship between single-channel conductance and *V*_h_, the single-channel conductance for each channel was plotted against *V*_h_ for the same channel in Fig. 6 C for the indicated channel types. A linear relationship was observed (Fig. 6 C; R = 0.86, P < 0.0001). When taken together, the data in Fig. 6, B and C, are consistent with the idea that replacing a WT subunit with a mutant subunit adds both an increment of negative voltage shift to *V*_h_ and a step decrease in single-channel conductance. Thus, at the single-channel level, the heterozygous G375R mutation acts simultaneously as a GOF mutation to shift voltage activation to more negative voltages (Figs. 4 and 5) and as a DOF mutation to decrease single-channel conductance (Fig. 6). At the whole-cell and macropatch level, the increase in currents from the GOF negative shift in activation would dominate the DOF reduction in single-channel conductance, producing large left-shifted currents (Figs. 2, 3, S2, and S3). The DOF in conductance would act to decrease the consequences of the negative shift in activation.

### Linear incremental model for the contributions of mutant and WT subunits to single-channel conductance

A linear incremental model in which each WT subunit contributes 76.5 pS to single channel conductance and each mutant subunit contributes 46.1 pS could approximate the single channel conductance for the five types of assembled channels (Fig. 6 D dashed line; model in the figure legend).

### WT, hybrid, and G375R homotetrameric mutant BK channels are expressed in the HEK293 cell expression system

**Figure 7.**
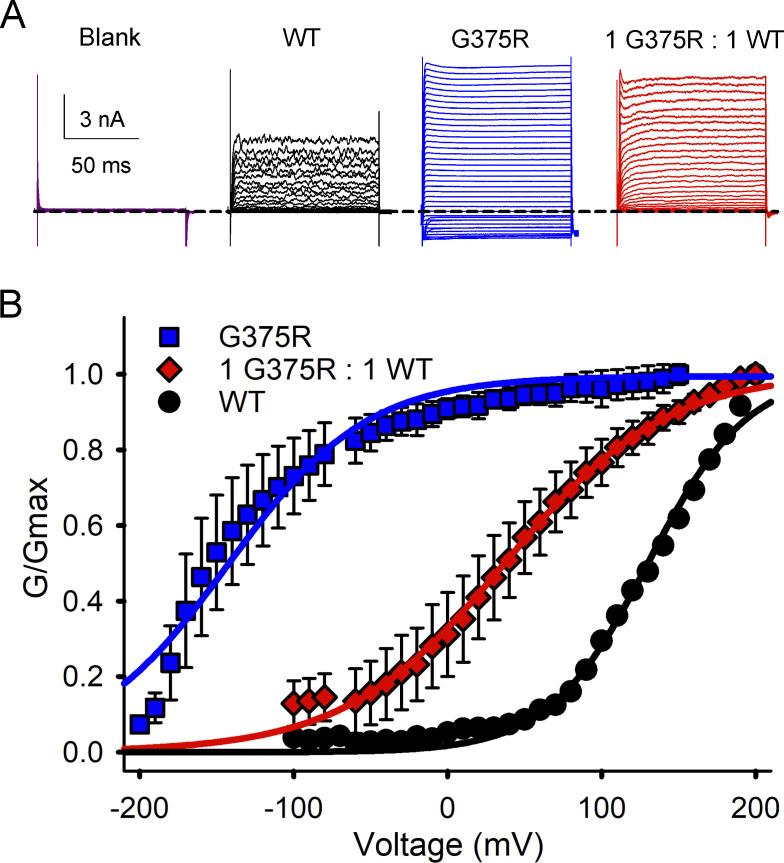
**G375R homotetrameric mutant channels, assembled channels, and WT BK channels are expressed in HEK293 cells. (A)** Whole-cell currents were recorded from HEK293 cells following transfection with the indicated types of cDNA. The whole-cell currents were generated by holding at −60 mV, with a prestep to −100 mV, followed by steps from either −200 or −100 to +200 mV in 10 mV increments followed by a post-step to −120 mV. The dashed line indicates the level of 0 current. Evoked currents were not observed in HEK293 cells that were not transfected. **(B)** Plots of normalized conductance versus the voltage of the activating steps following transfections with G375R mutant cDNA (blue squares); a 1:1 mixture of mutant and WT cDNA (red diamonds) to express assembled channels; or WT cDNA (black circles). Unlike single-channel recordings (Fig. 4), whole-cell recordings reflect the average response from many hundreds to thousands of channels. There were large negative shifts in *V*_h_ in the G-V curves determined from G375R homotetrameric mutant channels (blue) and from assembled channels (red) compared with WT channels (black). The mean *V*_h_ was 130.3 ± 4.4 mV (*n* = 4) for WT BK currents, 37.5 ± 2.7 mV (*n* = 5) for BK currents following the 1:1 injection, and −142.5 ± 3.6 mV (*n* = 3) for the G375R homotetrameric mutant channels. The 1:1 transfection also decreased the voltage sensitivity of activation compared with WT, with a slope of 52.0 ± 2.0 mV (*n* = 5) per e-fold change for current after a 1:1 transfection, compared with 32.3 ± 2.5 mV (*n* = 4) for WT currents. The G375R homotetrameric mutant channels had a slope of 45.0 ± 4.0 mV. Mean ± SEM.

Liang et al. (2019) reported that no potassium currents were recorded from excised macropatches following transfection of HEK293T cells with plasmids coding for G375R mutant BK subunits. In contrast, we found that functional single homotetrameric mutant channels were readily expressed following injection of *Xenopus* oocytes with cRNA coding for mutant BK G375R subunits (Fig. 4, A, and C). To examine if these differences in channel expression were due to differences in expression systems, we transfected HEK293 cells with WT cDNA to generate WT channels, with mutant G375R cDNA to generate homotetrameric mutant channels, and with a 1:1 mixture of WT and mutant cDNA to mimic the generation of channels that would normally arise from a heterozygous mutation. We found that the transfected HEK293 cells displayed robust whole-cell BK currents for each of the three different types of transfections (Fig. 7 A). The macroscopic G-V curves from HEK293 cells for transfection with WT cDNA alone or 1:1 transfection with WT and mutant cDNA were very similar to the macroscopic G-V curves we obtained using the *Xenopus* oocyte expression system (compare Fig. 7 with Fig. 3). We also found that the whole cell current from G375R homotetrameric mutant channels expressed in HEK293 cells had a *V*_h_ value within the broad range of *V*_h_ values of single-channel recordings from patches of membrane excised from *Xenopus* oocytes injected with G375R mutant mRNA (Fig. 4).

Hence, in both *Xenopus* oocytes and HEK293 expression systems, we observed functional G375R homotetrameric mutant currents that displayed a marked negative shift in activation consistent with a GOF mutation. This contrasts with the observations of Liang et al. (2019), who did not observe BK channel currents for G375R transfection of HEK293T cells. They reported G375R as a LOF mutation. The reason for the difference in observations is not known, but we found that the viability of *Xenopus* oocytes injected with G375R cRNA was reduced (see Materials and methods).

### Whole-cell and macropatch currents can obscure mechanism

The narrow error bars for the macro currents recorded from whole oocytes and excised macropatches (Figs. 2, 3, S2, and S3) following injection of WT cRNA or a 1:1 injection of mutant and WT cRNA, indicated that the mean responses recorded from hundreds to thousands of channels were repeatable. Narrow error bars for mean responses do not necessarily indicate that the individual channels underlying the mean response have near identical properties, as this was not the case for the macro currents following 1:1 injection of mutant and WT cRNA, where individual assembled channels following the 1:1 injection had a very wide range of *V*_h_ that spanned over 300 mV (Fig. 4 B). In spite of this wide range for individual channels, the mean G-V data following the 1:1 injections were reasonably well described by a single Boltzmann function (Fig. 3 B, blue line through red diamonds), but were better described (red line) by assuming five underlying channel types, as described in the legend to Fig. 3.

## Discussion

The heterozygous G375R BK channel variant has been associated with a devastating human phenotype that includes malformation syndrome and severe neurological and developmental disorders (Liang et al., 2019). This variant has only appeared in the human population when heterozygous with WT, perhaps because a homozygous G375R genotype may not permit viability because of the extreme GOF phenotype we observed for homotetrameric mutant channels. To gain insight into the pathogenicity of this variant, currents from whole cells, macropatches, and single channels were recorded from BK channels expressed following a 1:1 injection of G375R mutant and WT cRNA to mimic a G375R mutation heterozygous with WT.

Recordings from whole cells and macropatches, both of which contain many hundreds to thousands of channels, indicated that the *V*_h_ of the current following a 1:1 injection, was left shifted to more negative potentials by about −120 mV compared with WT currents (Figs. 2, 3, S2, and S3). The aberrant BK channels underlying the negative shifts in activation would lead to a much greater fraction of BK channels being open at negative membrane potentials, including at potentials near the resting potential (Figs. 2, 3, S2, and S3), which would oppose cellular depolarization, altering cellular function.

To explore the underlying molecular basis for the aberrant current activation associated with the heterozygous G375R variant, we used high-resolution single-channel recording to isolate and characterize the BK channels that are assembled and expressed following injection of a 1:1 mixture of G375R mutant and WT cRNA. Theoretical considerations based on equal production and a random assembly of mutant and WT subunits suggest there could be multiple types of assembled channels with different subunit combinations (MacKinnon, 1991; Blaine and Ribera, 1998; Bergendahl et al., 2019; Backwell and Marsh, 2022; Fig. 1). Consistent with this possibility, our analysis suggested that five different types of functional BK channels were expressed: 3% were consistent with WT, 12% with homotetrameric mutant, and 85% with three different types of hybrid channels of mixed subunits (Fig. 4; and Fig. 5, A and B). The percentages of expression of these five types of functionally assembled channels were not significantly different (Fig. S4) from the theoretical predictions of Fig. 1 based on equal production and a random assembly of subunits. This suggests, within the limits of experimental variability (Fig. S4), that the processes involved in subunit production, assembly, and expression do not distinguish significantly between mutant and WT alleles and subunits, so the phenotypic differences arise at the functional level of individual channels.

The three types of hybrid channels comprising 85% of the expressed assembled channels had properties falling between those of mutant and WT channels that varied with their apparent subunit composition (Figs. 4, 5, and 6). 97% of the assembled channels, all except for the 3% WT, displayed both GOF negative shifts in activation and smaller DOF reductions in single-channel conductance (Figs. 4, 5, and 6). Hence, most of the channels expressed for the heterozygous G375R mutation displayed aberrant properties. The values of *V*_h_ and single channel conductance for each of the five types of functional assembled channels could be predicted with a linear incremental model in which each mutant and WT subunit in each of the five types of functional assembled channels acted relatively independently to contribute increments of both *V*_h_ and single-channel conductance to the molecular phenotype of the channel (Fig. 5 C and Fig. 6 D; the models are in the figure legends).

A potential mechanism to produce the five functional types of assembled channels for a heterozygous G375R BK channel mutation, then, is equal production and a random assembly of mutant and WT subunits into channels of five different subunit compositions, where the *V*_h_ and single-channel conductance of each channel type are determined by independent contributions from each of the four subunits in a channel. Each WT subunit adds increments of 40.5 mV to *V*_h_ and 76.5 pS to single-channel conductance, and each mutant subunit adds increments of −16.7 mV to *V*_h_ and 46.1 pS to single-channel conductance (Fig. 5 C and Fig. 6). Whereas a linear incremental model could provide reasonable descriptions of the data, further study is likely to reveal added complexity.

We were surprised to find that the mean macroscopic G-V curve following a 1:1 injection of G375R mutant and WT cRNA could also be well described by a single Boltzmann function (Fig. 3) as the G-V curve arose from the sum of currents from five different types of BK channels with markedly different properties (Figs. 4, 5, and 6). Consequently, an observation that a macroscopic G-V curve is well described by a single Boltzmann function does not necessarily exclude the possibility that the macroscopic G-V curve arises instead from multiple types of channels with different properties. The description with a single Boltzmann function may be possible in this case because the percentages of the five types of contributing channels first increase and then decrease (Fig. 1 and Fig. 5 B), helping to fill in and smooth the G-V curve. Paradoxically, the single-Boltzmann shape of the G-V curves for 1:1 injections and transfections (Fig. 3 and Fig. 7) provides additional evidence for the assembly and expression of hybrid channels, as there would be two clearly separable Boltzmann components in the 1:1 G-V curves arising from homotetrameric mutant and WT channels if the G375R and WT subunits did not coassemble to form three types of hybrid channels with intermediate properties to fill in and smooth the G-V curve.

For classification with regard to genetic disease (Backwell and Marsh, 2022), we suggest that the heterozygous G375R mutation be labeled as an assembly-mediated dominant GOF mutation: assembly-mediated to indicate that mutant and WT subunits assemble into multiple types of functional tetrameric BK channels, dominant because assembled channels with one or more mutant subunits display pathogenic properties, and GOF because less depolarization is required to activate BK channels with one or more mutant subunits. The net result is that 94–97% of the expressed channels display aberrant activation (Figs. 1, 4, and 5). A dominant GOF phenotype has been described previously for the heterozygous G88R mutation of the TASK-4 K^+^ channel in the heart, based on a study using whole-cell currents (Friedrich et al., 2014).

An assembly-mediated dominant-GOF mutation for heterozygous G375R can be compared to the well-known dominant-negative effect observed for some types of protein complexes and channelopathies (Fink et al., 1996; Ribera et al., 1996; Silberberg et al., 2005; Reed et al., 2016; Du et al., 2020; Hichri et al., 2020; Backwell and Marsh, 2022). Both can cause disease through a disproportionate fraction of the channels affected. In the first case, there is a GOF in channels with one or more mutant subunits, and in the second there is a complete or partial loss of function of channels associated with, typically, one or more mutant subunits.

Whereas an assembly-mediated dominant GOF classification of the heterozygous G375R mutation is useful to suggest the potential underlying basis of genetic disease, as it describes the net functional cellular phenotype, the molecular phenotype is more complex. The action of the heterozygous G375R mutation is to generate five types of channels, four of which simultaneously display a GOF in activation and a smaller DOF in single-channel conduction. The GOF dominates the response at the level of cellular currents.

The random assembly of mutant and WT subunits into tetrameric BK channels when mimicking a heterozygous mutation led to multiple types of genetic dominance at the level of the molecular phenotype when viewed with single-channel recording. Codominance was observed for the WT and homotetrameric mutant channels, and partial dominance was observed for the three types of hybrid channels. Support for codominance was that the homotetrameric mutant and WT channels expressed in the molecular phenotype when mimicking a heterozygous mutation had the same molecular phenotypes as homotetrameric mutant and WT channels expressed following injection of only mutant or WT cRNA (Figs. 4, 5, and 6). Support for partial dominance was that the three types of expressed hybrid channels had individual values of *V*_h_ and single channel conductance that were distinct from each other and fell in the range between those of homotetrameric mutant and WT channels (Fig. 5 C and Fig. 6 D). The levels of partial dominance in the linear incremental model (Fig. 5 C and Fig. 6 D) were determined by the numbers of mutant and WT subunits per channel acting independently of one another, rather than by mutant subunits altering the function of WT subunits, as in some classical descriptions of genetic dominance. It is remarkable that a single base pair substitution in one allele of a pair of alleles that encode for the α subunit of BK channels results in the expression of WT BK channels plus four aberrant types of BK channels, each with different molecular phenotypes that simultaneously display a dominant GOF in *V*_h_ and a lessor DOF in single-channel conductance, with the five types of functional channels displaying either codominance or one of three levels of partial dominance for both *V*_h_ and single channel conductance.

How do our observations of independent interactions of the G375R mutant and WT pore-forming subunits compare to interactions of these subunits with regulatory subunits? Wang et al. (2002) found that single BK channels comprised of four subunits could be associated with zero to four regulatory β2-subunits per channel, with each β2-subunit giving incremental changes in *V*_h_. Hence, both mutated and WT pore-forming subunits (Fig. 5 C; Niu and Magleby, 2002) and the non-pore-forming regulatory β2-subunits (Wang et al., 2002) can give incremental changes in gating, but such incremental changes per subunit are not necessarily universal, so each type of subunit will need to be assessed. A single BK channel comprised of four pore-forming subunits can also include up to four regulatory γ1-subunits, but a single γ1-subunit per channel is sufficient to induce the full gating shift induced by γ1-subunits (Gonzalez-Perez et al., 2014, 2018).

Devising therapies for the de novo G375R heterozygous mutation will be challenging. Theoretical and experimental observations (Figs. 1, 4, and 5) suggest that most (94–97%) of the channels for a mutation heterozygous with WT would be pathogenic, with only 3–6% of the channels WT. The pathogenic channels would consist of multiple channel types, each with large differences in activation properties and smaller differences in conductance (Figs. 4, 5, and 6). Therapies to block or inactivate the pathogenic channels would ideally silence the multiple types of pathogenic channels while leaving any WT channels intact. Even if such selective blockers could be devised, it is unlikely that the remaining 3–6% of WT channels would be sufficient to restore normal cellular function. Effective therapies will likely require replacing or silencing the mutant alleles or preventing mutant subunits from assembling with themselves and WT subunits if/when such techniques become practical in humans.

## References

[bib1] Backwell, L., and J.A. Marsh. 2022. Diverse molecular mechanisms underlying pathogenic protein mutations: Beyond the loss-of-function paradigm. Annu. Rev. Genomics Hum. Genet. 23:475–498. 10.1146/annurev-genom-111221-103208 35395171

[bib2] Bailey, C.S., H.J. Moldenhauer, S.M. Park, S. Keros, and A.L. Meredith. 2019. KCNMA1-linked channelopathy. J. Gen. Physiol. 151:1173–1189. 10.1085/jgp.201912457 31427379 PMC6785733

[bib3] Barrett, J.N., K.L. Magleby, and B.S. Pallotta. 1982. Properties of single calcium-activated potassium channels in cultured rat muscle. J. Physiol. 331:211–230. 10.1113/jphysiol.1982.sp014370 6296366 PMC1197747

[bib4] Bergendahl, L.T., L. Gerasimavicius, J. Miles, L. Macdonald, J.N. Wells, J.P.I. Welburn, and J.A. Marsh. 2019. The role of protein complexes in human genetic disease. Protein Sci. 28:1400–1411. 10.1002/pro.3667 31219644 PMC6635777

[bib5] Blaine, J.T., and A.B. Ribera. 1998. Heteromultimeric potassium channels formed by members of the Kv2 subfamily. J. Neurosci. 18:9585–9593. 10.1523/JNEUROSCI.18-23-09585.1998 9822719 PMC6793320

[bib6] Brelidze, T.I., X. Niu, and K.L. Magleby. 2003. A ring of eight conserved negatively charged amino acids doubles the conductance of BK channels and prevents inward rectification. Proc. Natl. Acad. Sci. USA. 100:9017–9022. 10.1073/pnas.1532257100 12843404 PMC166430

[bib7] Brenner, R., G.J. Peréz, A.D. Bonev, D.M. Eckman, J.C. Kosek, S.W. Wiler, A.J. Patterson, M.T. Nelson, and R.W. Aldrich. 2000. Vasoregulation by the β1 subunit of the calcium-activated potassium channel. Nature. 407:870–876. 10.1038/35038011 11057658

[bib8] Chen, X., J. Yan, and R.W. Aldrich. 2014. BK channel opening involves side-chain reorientation of multiple deep-pore residues. Proc. Natl. Acad. Sci. USA. 111:E79–E88. 10.1073/pnas.1321697111 24367115 PMC3890798

[bib9] Colquhoun, D. 1971. Lectures on Biostatistics. Clarendon Press, Oxford. pp. 64–115.

[bib10] Cui, J. 2021. BK channel gating mechanisms: Progresses toward a better understanding of variants linked neurological diseases. Front. Physiol. 12:762175. 10.3389/fphys.2021.762175 34744799 PMC8567085

[bib11] Ding, J.P., Z.W. Li, and C.J. Lingle. 1998. Inactivating BK channels in rat chromaffin cells may arise from heteromultimeric assembly of distinct inactivation-competent and noninactivating subunits. Biophys. J. 74:268–289. 10.1016/S0006-3495(98)77785-9 9449328 PMC1299380

[bib12] Du, X., J.L. Carvalho-de-Souza, C. Wei, W. Carrasquel-Ursulaez, Y. Lorenzo, N. Gonzalez, T. Kubota, J. Staisch, T. Hain, N. Petrossian, . 2020. Loss-of-function BK channel mutation causes impaired mitochondria and progressive cerebellar ataxia. Proc. Natl. Acad. Sci. USA. 117:6023–6034. 10.1073/pnas.1920008117 32132200 PMC7084159

[bib13] Fink, M., F. Duprat, C. Heurteaux, F. Lesage, G. Romey, J. Barhanin, and M. Lazdunski. 1996. Dominant negative chimeras provide evidence for homo and heteromultimeric assembly of inward rectifier K^+^ channel proteins via their N-terminal end. FEBS Lett. 378:64–68. 10.1016/0014-5793(95)01388-1 8549804

[bib14] Friedrich, C., S. Rinné, S. Zumhagen, A.K. Kiper, N. Silbernagel, M.F. Netter, B. Stallmeyer, E. Schulze-Bahr, and N. Decher. 2014. Gain-of-function mutation in TASK-4 channels and severe cardiac conduction disorder. EMBO Mol. Med. 6:937–951. 10.15252/emmm.201303783 24972929 PMC4119356

[bib15] Geng, Y., Z. Deng, G. Zhang, G. Budelli, A. Butler, P. Yuan, J. Cui, L. Salkoff, and K.L. Magleby. 2020. Coupling of Ca^2+^ and voltage activation in BK channels through the αB helix/voltage sensor interface. Proc. Natl. Acad. Sci. USA. 117:14512–14521. 10.1073/pnas.1908183117 32513714 PMC7321994

[bib16] Geng, Y., and K.L. Magleby. 2015. Single-channel kinetics of BK (Slo1) channels. Front. Physiol. 5:532. 10.3389/fphys.2014.00532 25653620 PMC4300911

[bib17] Geng, Y., X. Niu, and K.L. Magleby. 2011. Low resistance, large dimension entrance to the inner cavity of BK channels determined by changing side-chain volume. J. Gen. Physiol. 137:533–548. 10.1085/jgp.201110616 21576375 PMC3105516

[bib18] Gonzalez-Perez, V., M. Ben Johny, X.M. Xia, and C.J. Lingle. 2018. Regulatory γ1 subunits defy symmetry in functional modulation of BK channels. Proc. Natl. Acad. Sci. USA. 115:9923–9928. 10.1073/pnas.1804560115 30224470 PMC6176617

[bib19] Gonzalez-Perez, V., X.M. Xia, and C.J. Lingle. 2014. Functional regulation of BK potassium channels by γ1 auxiliary subunits. Proc. Natl. Acad. Sci. USA. 111:4868–4873. 10.1073/pnas.1322123111 24639523 PMC3977294

[bib20] Gonzalez-Perez, V., X.M. Xia, and C.J. Lingle. 2015. Two classes of regulatory subunits coassemble in the same BK channel and independently regulate gating. Nat. Commun. 6:8341. 10.1038/ncomms9341 26388335 PMC4578311

[bib21] Gu, N., K. Vervaeke, and J.F. Storm. 2007. BK potassium channels facilitate high-frequency firing and cause early spike frequency adaptation in rat CA1 hippocampal pyramidal cells. J. Physiol. 580:859–882. 10.1113/jphysiol.2006.126367 17303637 PMC2075463

[bib22] Hamill, O.P., A. Marty, E. Neher, B. Sakmann, and F.J. Sigworth. 1981. Improved patch-clamp techniques for high-resolution current recording from cells and cell-free membrane patches. Pflugers Arch. 391:85–100. 10.1007/BF00656997 6270629

[bib23] Han, Y., P. Li, and M.M. Slaughter. 2004. Selective antagonism of rat inhibitory glycine receptor subunits. J. Physiol. 554:649–658. 10.1113/jphysiol.2003.056309 14645455 PMC1664810

[bib24] Harvey, J.R.M., A.E. Plante, and A.L. Meredith. 2020. Ion channels controlling circadian rhythms in suprachiasmatic nucleus excitability. Physiol. Rev. 100:1415–1454. 10.1152/physrev.00027.2019 32163720 PMC7717126

[bib25] Hichri, E., Z. Selimi, and J.P. Kucera. 2020. Modeling the interactions between sodium channels provides insight into the negative dominance of certain channel mutations. Front. Physiol. 11:589386. 10.3389/fphys.2020.589386 33250780 PMC7674773

[bib26] Hite, R.K., X. Tao, and R. MacKinnon. 2017. Structural basis for gating the high-conductance Ca^2+^-activated K^+^ channel. Nature. 541:52–57. 10.1038/nature20775 27974801 PMC5513477

[bib27] Horrigan, F.T. 2012. Perspectives on: Conformational coupling in ion channels: Conformational coupling in BK potassium channels. J. Gen. Physiol. 140:625–634. 10.1085/jgp.201210849 23183698 PMC3514727

[bib28] Horrigan, F.T., and R.W. Aldrich. 2002. Coupling between voltage sensor activation, Ca^2+^ binding and channel opening in large conductance (BK) potassium channels. J. Gen. Physiol. 120:267–305. 10.1085/jgp.20028605 12198087 PMC2229516

[bib29] Latorre, R., K. Castillo, W. Carrasquel-Ursulaez, R.V. Sepulveda, F. Gonzalez-Nilo, C. Gonzalez, and O. Alvarez. 2017. Molecular determinants of BK channel functional diversity and functioning. Physiol. Rev. 97:39–87. 10.1152/physrev.00001.2016 27807200

[bib30] Latorre, R., C. Vergara, and C. Hidalgo. 1982. Reconstitution in planar lipid bilayers of a Ca^2+^-dependent K^+^ channel from transverse tubule membranes isolated from rabbit skeletal muscle. Proc. Natl. Acad. Sci. USA. 79:805–809. 10.1073/pnas.79.3.805 6278496 PMC345841

[bib31] Liang, L., X. Li, S. Moutton, S.A. Schrier Vergano, B. Cogné, A. Saint-Martin, A.C.E. Hurst, Y. Hu, O. Bodamer, J. Thevenon, . 2019. De novo loss-of-function KCNMA1 variants are associated with a new multiple malformation syndrome and a broad spectrum of developmental and neurological phenotypes. Hum. Mol. Genet. 28:2937–2951. 10.1093/hmg/ddz117 31152168 PMC6735855

[bib32] MacKinnon, R. 1991. Determination of the subunit stoichiometry of a voltage-activated potassium channel. Nature. 350:232–235. 10.1038/350232a0 1706481

[bib33] McCobb, D.P., N.L. Fowler, T. Featherstone, C.J. Lingle, M. Saito, J.E. Krause, and L. Salkoff. 1995. A human calcium-activated potassium channel gene expressed in vascular smooth muscle. Am. J. Physiol. 269:H767–H777. 10.1152/ajpheart.1995.269.3.H767 7573516

[bib34] McManus, O.B., and K.L. Magleby. 1991. Accounting for the Ca^2+^-dependent kinetics of single large-conductance Ca^2+^-activated K^+^ channels in rat skeletal muscle. J. Physiol. 443:739–777. 10.1113/jphysiol.1991.sp018861 1822543 PMC1179869

[bib35] Miller, D.C., M.M. Weinstock, and K.L. Magleby. 1978. Is the quantum of transmitter release composed of subunits? Nature. 274:388–390. 10.1038/274388a0 27723

[bib36] Miller, J.P., H.J. Moldenhauer, S. Keros, and A.L. Meredith. 2021. An emerging spectrum of variants and clinical features in KCNMA1-linked channelopathy. Channels. 15:447–464. 10.1080/19336950.2021.1938852 34224328 PMC8259716

[bib37] Montgomery, J.R., and A.L. Meredith. 2012. Genetic activation of BK currents in vivo generates bidirectional effects on neuronal excitability. Proc. Natl. Acad. Sci. USA. 109:18997–19002. 10.1073/pnas.1205573109 23112153 PMC3503162

[bib38] Nimigean, C.M., J.S. Chappie, and C. Miller. 2003. Electrostatic tuning of ion conductance in potassium channels. Biochemistry. 42:9263–9268. 10.1021/bi0348720 12899612

[bib39] Niu, X., and K.L. Magleby. 2002. Stepwise contribution of each subunit to the cooperative activation of BK channels by Ca^2+^. Proc. Natl. Acad. Sci. USA. 99:11441–11446. 10.1073/pnas.172254699 12161564 PMC123275

[bib40] Pantazis, A., and R. Olcese. 2016. Biophysics of BK channel gating. Int. Rev. Neurobiol. 128:1–49. 10.1016/bs.irn.2016.03.013 27238260

[bib41] Park, S.M., C.E. Roache, P.H. Iffland II, H.J. Moldenhauer, K.K. Matychak, A.E. Plante, A.G. Lieberman, P.B. Crino, and A. Meredith. 2022. BK channel properties correlate with neurobehavioCral severity in three KCNMA1-linked channelopathy mouse models. Elife. 11:11. 10.7554/eLife.77953 PMC9275823 35819138

[bib42] Reed, A.P., G. Bucci, F. Abd-Wahab, and S.J. Tucker. 2016. Dominant-negative effect of a missense variant in the TASK-2 (KCNK5) K^+^ channel associated with balkan endemic nephropathy. PLoS One. 11:e0156456. 10.1371/journal.pone.0156456 27228168 PMC4882002

[bib43] Ribera, A.B., L.M. Pacioretty, and R.S. Taylor. 1996. Probing molecular identity of native single potassium channels by overexpression of dominant negative subunits. Neuropharmacology. 35:1007–1016. 10.1016/0028-3908(96)00098-6 8938731

[bib44] Robitaille, R., M.L. Garcia, G.J. Kaczorowski, and M.P. Charlton. 1993. Functional colocalization of calcium and calcium-gated potassium channels in control of transmitter release. Neuron. 11:645–655. 10.1016/0896-6273(93)90076-4 7691106

[bib45] Rothberg, B.S., and K.L. Magleby. 2000. Voltage and Ca^2+^ activation of single large-conductance Ca^2+^-activated K^+^ channels described by a two-tiered allosteric gating mechanism. J. Gen. Physiol. 116:75–99. 10.1085/jgp.116.1.75 10871641 PMC2229615

[bib46] Salkoff, L., A. Butler, G. Ferreira, C. Santi, and A. Wei. 2006. High-conductance potassium channels of the SLO family. Nat. Rev. Neurosci. 7:921–931. 10.1038/nrn1992 17115074

[bib47] Santi, C.M., G. Ferreira, B. Yang, V.R. Gazula, A. Butler, A. Wei, L.K. Kaczmarek, and L. Salkoff. 2006. Opposite regulation of Slick and Slack K^+^ channels by neuromodulators. J. Neurosci. 26:5059–5068. 10.1523/JNEUROSCI.3372-05.2006 16687497 PMC6674240

[bib48] Silberberg, S.D., T.H. Chang, and K.J. Swartz. 2005. Secondary structure and gating rearrangements of transmembrane segments in rat P2X4 receptor channels. J. Gen. Physiol. 125:347–359. 10.1085/jgp.200409221 15795310 PMC2217512

[bib49] Tao, X., R.K. Hite, and R. MacKinnon. 2017. Cryo-EM structure of the open high-conductance Ca^2+^-activated K^+^ channel. Nature. 541:46–51. 10.1038/nature20608 27974795 PMC5500982

[bib50] Tao, X., and R. MacKinnon. 2019. Molecular structures of the human Slo1 K^+^ channel in complex with β4. Elife. 8:8. 10.7554/eLife.51409 PMC6934384 31815672

[bib51] Wang, B., B.S. Rothberg, and R. Brenner. 2009. Mechanism of increased BK channel activation from a channel mutation that causes epilepsy. J. Gen. Physiol. 133:283–294. 10.1085/jgp.200810141 19204188 PMC2654085

[bib52] Wang, Y.W., J.P. Ding, X.M. Xia, and C.J. Lingle. 2002. Consequences of the stoichiometry of *Slo1* α and auxiliary β subunits on functional properties of large-conductance Ca^2+^-activated K^+^ channels. J. Neurosci. 22:1550–1561. 10.1523/JNEUROSCI.22-05-01550.2002 11880485 PMC6758889

[bib53] Xia, X.M., X. Zeng, and C.J. Lingle. 2002. Multiple regulatory sites in large-conductance calcium-activated potassium channels. Nature. 418:880–884. 10.1038/nature00956 12192411

[bib54] Yang, J., G. Krishnamoorthy, A. Saxena, G. Zhang, J. Shi, H. Yang, K. Delaloye, D. Sept, and J. Cui. 2010. An epilepsy/dyskinesia-associated mutation enhances BK channel activation by potentiating Ca^2+^ sensing. Neuron. 66:871–883. 10.1016/j.neuron.2010.05.009 20620873 PMC2907746

[bib55] Zhou, Y., H. Yang, J. Cui, and C.J. Lingle. 2017. Threading the biophysics of mammalian Slo1 channels onto structures of an invertebrate Slo1 channel. J. Gen. Physiol. 149:985–1007. 10.1085/jgp.201711845 29025867 PMC5677106

